# The hidden diversity of ancient bornaviral sequences from X and P genes in vertebrate genomes

**DOI:** 10.1093/ve/vead038

**Published:** 2023-06-03

**Authors:** Bea Clarise B Garcia, Yahiro Mukai, Keizo Tomonaga, Masayuki Horie

**Affiliations:** Laboratory of Veterinary Microbiology, Graduate School of Veterinary Science, Osaka Metropolitan University, 1-58 Rinku Orai-kita, Izumisano, Osaka 598-8531, Japan; Laboratory of RNA Viruses, Institute for Frontier Life and Medical Sciences, Kyoto University, 53 Kawahara-cho, Shogo-in, Sakyo, Kyoto 606-8507, Japan; Laboratory of RNA Viruses, Department of Mammalian Regulatory Network, Graduate School of Biostudies, Kyoto University, 53 Kawahara-cho, Shogo-in, Sakyo, Kyoto 606-8507, Japan; Laboratory of RNA Viruses, Institute for Frontier Life and Medical Sciences, Kyoto University, 53 Kawahara-cho, Shogo-in, Sakyo, Kyoto 606-8507, Japan; Laboratory of RNA Viruses, Department of Mammalian Regulatory Network, Graduate School of Biostudies, Kyoto University, 53 Kawahara-cho, Shogo-in, Sakyo, Kyoto 606-8507, Japan; Department of Molecular Virology, Graduate School of Medicine, Kyoto University, 53 Kawahara-cho, Shogo-in, Sakyo, Kyoto 606-8507, Japan; Laboratory of Veterinary Microbiology, Graduate School of Veterinary Science, Osaka Metropolitan University, 1-58 Rinku Orai-kita, Izumisano, Osaka 598-8531, Japan; Osaka International Research Center for Infectious Diseases, Osaka Metropolitan University, 1-58 Rinku Orai-kita, Izumisano, Osaka 598-8531, Japan

**Keywords:** endogenous viral elements, endogenous bornavirus-like elements, bornavirus, read-through transcript, fusion gene

## Abstract

Endogenous bornavirus–like elements (EBLs) are heritable sequences derived from bornaviruses in vertebrate genomes that originate from transcripts of ancient bornaviruses. EBLs have been detected using sequence similarity searches such as tBLASTn, whose technical limitations may hinder the detection of EBLs derived from small and/or rapidly evolving viral X and P genes. Indeed, no EBLs derived from the X and P genes of orthobornaviruses have been detected to date in vertebrate genomes. Here, we aimed to develop a novel strategy to detect such ‘hidden’ EBLs. To this aim, we focused on the 1.9-kb read-through transcript of orthobornaviruses, which encodes a well-conserved N gene and small and rapidly evolving X and P genes. We show a series of evidence supporting the existence of EBLs derived from orthobornaviral X and P genes (EBLX/Ps) in mammalian genomes. Furthermore, we found that an EBLX/P is expressed as a fusion transcript with the cellular gene, *ZNF451*, which potentially encodes the ZNF451/EBLP fusion protein in miniopterid bat cells. This study contributes to a deeper understanding of ancient bornaviruses and co-evolution between bornaviruses and their hosts. Furthermore, our data suggest that endogenous viral elements are more abundant than those previously appreciated using BLAST searches alone, and further studies are required to understand ancient viruses more accurately.

## Introduction

Ancient viral sequences can be found within the genomes of organisms in the form of endogenous viral elements (EVEs; Aiewsakun and Katzourakis 2015). EVEs are derived from the integration of viral sequences into the host germline DNA and transfer of the integrated sequences to subsequent progenies (Jern and Coffin 2008; Aiewsakun and Katzourakis 2015). As molecular fossils of ancient viruses, EVEs have expanded our knowledge of the diversity, host range, and long-term evolution of viruses (Aiewsakun and Katzourakis 2015). Furthermore, some EVEs are co-opted by hosts to function as novel gene regulatory elements, non-coding RNAs, and proteins (Jern and Coffin 2008).

Most EVEs are derived from retroviruses, as the viruses reverse-transcribe and integrate the viral genomes into the host genomes using virally encoded enzymes. However, non-retroviral EVEs have also been identified. Endogenous bornavirus–like elements (EBLs) are EVEs derived from bornaviruses (family *Bornaviridae*), a group of non-segmented negative-strand RNA viruses (Horie et al. 2010; Rubbenstroth et al. 2021). Although bornaviruses are RNA viruses, evidence strongly suggests that bornaviral mRNA is reverse-transcribed and integrated into the host genome via the activity of the long interspersed nuclear element 1 (LINE-1) retrotransposon (Belyi, Levine, and Skalka 2010; Horie et al. 2010). We previously revealed an abundance of EBLs in vertebrate genomes derived from the bornaviral genes N (nucleoprotein), P (phosphoprotein), M (matrix protein), G (envelope glycoprotein), and L (large RNA-dependent RNA polymerase) (Fig. S1), which are referred to as EBLN, EBLP, EBLM, EBLG, and EBLL, respectively (Kawasaki et al. 2021). These EBLs originated from ancient bornaviruses of all genera in the family *Bornaviridae: Orthobornavirus, Carbovirus*, and *Cultervirus*.

**Figure 1.**
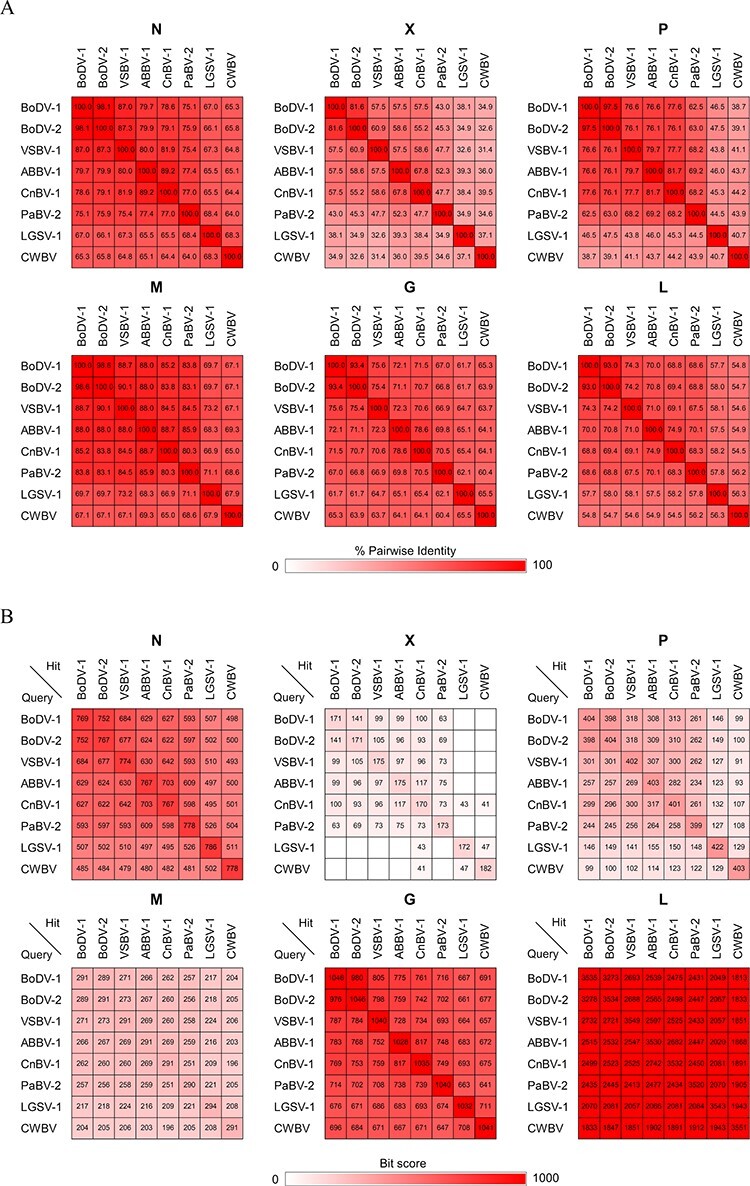
Conservation of amino acid sequences between extant orthobornaviral proteins. (A) Pairwise identities between the amino acid sequences of extant orthobornaviral proteins were analyzed using the Sequence Demarcation Tool (Muhire, Varsani, and Martin 2014). (B) BLAST bit scores obtained by BLASTp searches. BLASTp searches were performed against the NCBI nt database using the viral sequences displayed on the left side of the heatmap tables as queries. The analyzed viruses, which were selected as representative from each clade of the orthobornavirus phylogenetic tree (Rubbenstroth et al. 2021), are listed in Table S9.

Despite the rigorous search, there has been a bias in the detection of EBLs. EBLNs comprise the majority of the detected EBLs, while no EBLs derived from the X and P genes have been reported except for a few carboviral EBLPs (Horie et al. 2010; Katzourakis, Gifford, and Malik 2010; Horie et al. 2013; Gilbert et al. 2014; Horie and Tomonaga 2019; Kawasaki et al. 2021). We proposed two hypotheses to explain the lack of EBLX and EBLP. First, X/P mRNA, which encodes the X and P proteins, may have a low propensity for reverse transcription and/or integration into the host genome. However, previous studies of Borna disease virus 1 (BoDV-1; genus *Orthobornavirus*) showed that X/P mRNA is the most abundant transcript among the viral mRNAs (Poenisch et al. 2008; Kojima et al. 2019), which should therefore provide enough chances for integration. Moreover, we have previously demonstrated that the X/P mRNA of BoDV-1 can be reverse-transcribed and integrated into the genome of host cells (Horie et al. 2010, 2013). This led us to the second hypothesis, which states that EBLs derived from the X and P genes are present but are difficult to detect due to technical limitations of the sequence similarity search. EBLs have been routinely searched using tBLASTn; however, this can be an insensitive method for detecting small and rapidly evolving genes (Horie and Tomonaga 2019). Indeed, the X and P genes are small and the least conserved among bornaviral genes (Fig. 1), probably due to their intrinsic nature as antagonists of antiviral immunity (Unterstab et al. 2005; Duggal and Emerman 2012; Li et al. 2013). If the second hypothesis is correct, some EBLs may remain undetected. Therefore, we could be missing important insights into ancient bornaviruses and the co-evolution of bornaviruses and their hosts.

In this study, we sought to develop a novel strategy to detect these hidden EBLs by focusing on the nature of the read-through transcription of orthobornaviruses that produce a transcript encoding a highly conserved N gene and rapidly evolving X and P genes. We show evidence supporting the existence of several EBLX/Ps in vertebrate genomes. Furthermore, an EBLX/P in miniopterid bats appears to be transcribed as chimeric transcripts with a host gene, suggesting a potential co-option of EBLs by the host.

## Results

### Miniopterid bat EBLN is possibly derived from an N/X/P transcript

**Figure 2.**
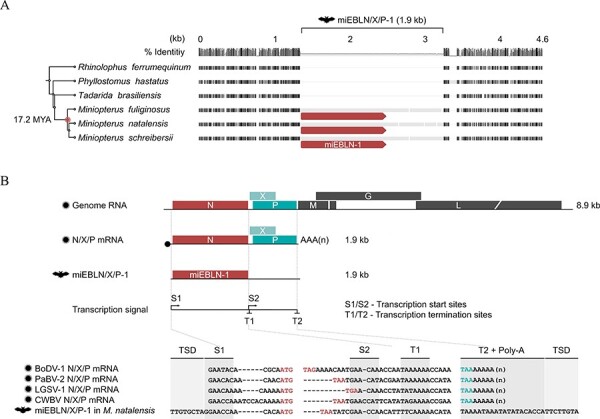
Characterization of miEBLN-1 as an EBL derived from N/X/P transcript. (A) Gene orthology analysis of the miEBLN/X/P-1 locus. Multiple alignment of the deduced syntenic loci between the indicated species is shown. The black boxes show conserved residues. The thin gray lines indicate alignment gaps. Species phylogeny and minimum integration age of miEBLN/X/P-1 are indicated on the left based on TimeTree (Kumar et al. 2017). The red arrow boxes indicate miEBLN-1 ORF. The full version of this alignment is available in Fig. S2a. (B) The upper panel shows the schematic diagram of orthobornaviral genomic RNA, 1.9-kb N/X/P mRNA, and miEBLN/X/P-1 and location of transcription signals (S1, S2, T1, and T2). The lower panel shows the nucleotide sequence alignment of extant orthobornaviral N/X/P mRNA and miEBLN/X/P-1. Red letters indicate the start and stop codons of the N gene. Teal blue letters indicate the stop codon of P gene. The full version of this alignment is available in Fig. S3.

We previously identified miEBLN-1, an EBLN derived from an ancient orthobornavirus that retains an intact N open reading frame (ORF) in miniopterid bats (Mukai et al. 2022). The miEBLN-1 locus is present in the genomes of miniopterid bats, specifically in those of *Miniopterus natalensis*, *Miniopterus schreibersii*, and *Miniopterus fuliginosus*, but is empty (i.e. syntenic loci lacking miEBLN-1) in the genomes of *Rhinolophus ferrumequinum* and *Phyllostomus hastatus*. We re-examined the presence of miEBLN-1 using the current genome datasets and found the miEBLN-1-empty locus in *Tadarida brasiliensis* (Figs. 2A and S2A).

Based on the nucleotide (nt) alignment of syntenic loci (Figs. 2A and S2A), we deduced the insertion size of the miEBLN-1 locus to be 1.9 kb, consisting of a 1.2-kb miEBLN-1 region and a 0.7-kb unannotated region. Therefore, we carried out a series of analyses to understand the source of the 0.7-kb unannotated region. First, we performed a BLASTn search (Camacho et al. 2009) against the NCBI nt database (Coordinators 2018) using the nt sequence of the 0.7-kb unannotated region as a query. The hits consisted only of miEBLN-1 sequences (data not shown). Next, we assessed whether the 0.7-kb unannotated region is derived from a short interspersed nuclear element (SINE) due to the precedence of chimeric integration comprising nt sequences from an N transcript and SINE (Belyi, Levine, and Skalka 2010; Horie et al. 2010). We performed a BLASTn search against the miniopterid bat genomes using the nt sequence of the 0.7-kb region as a query. We only detected five BLAST hits in the genomes of *M. natalensis* and *M. schreibersii*, which we identified as EBLs in the subsequent sections. We also analyzed the sequence of the 0.7-kb region by CENSOR (Jurka et al. 1996), but no hits were detected. These results indicated that the 0.7-kb fragment is not derived from a SINE.

We noticed that the insertion, including miEBLN-1, is almost similar in size to the N/X/P mRNA of extant orthobornaviruses (Figs. 2A and S2A), which led us to speculate that the insertion may be derived from an N/X/P transcript. N/X/P mRNA is produced by read-through of the termination signal T1, resulting in a 1.9-kb mRNA containing the transcription signal sequences S1, T1/S2, and T2 (Figs. 2B and S1) (Poenisch et al. 2008). Therefore, we investigated the presence of these signal-like sequences in the 1.9-kb miEBLN-1 insertion. We found transcription signal-like sequences at reasonable positions with respect to extant N/X/P transcripts (Figs. 2B and S3). A poly-A stretch was also observed immediately downstream of the T2 signal, indicating that the insertion originated from an mRNA transcript (Figs. 2B and S3). Furthermore, a tandem duplication sequence, TTGTGCTA/TTCTTGTA, was observed immediately upstream and downstream of S1 and T2-poly-A sequences, respectively (Figs. 2B and S3). This is a potential target site duplication (TSD), which is a hallmark of LINE-1-mediated mRNA integration (Morrish et al. 2002). These results suggested that the 1.9-kb insertion, including miEBLN-1, is derived from an ancient N/X/P transcript integrated by LINE-1. Therefore, we tentatively refer to this locus as miEBLN/X/P-1.

### Additional EBLs possibly derived from N/X/P transcripts in miniopterid bat genomes

**Figure 3.**
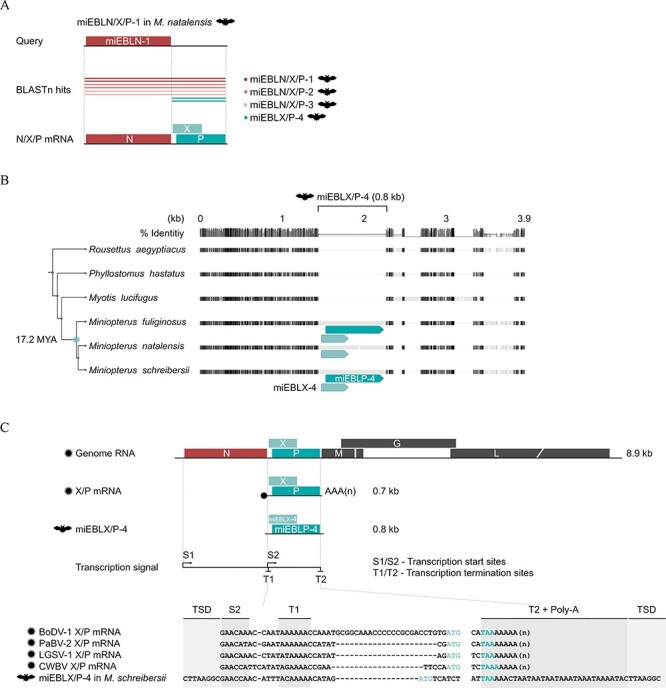
Identification of an EBL derived from X/P transcript in miniopterid bats. (A) Schematic diagram of the BLASTn search against the bat whole genome shotgun sequence database, using miEBLN/X/P-1 as a query. The BLAST hits are shown by dark to light read or teal blue lines corresponding to the EBLs indicated on the right. (B) Gene orthology analysis of the miEBLX/P-4 locus. Species phylogeny and deduced minimum integration age of miEBLX/P-4 are indicated on the left based on TimeTree (Kumar et al. 2017). miEBLX-4 and miEBLP-4 ORFs are shown in light and dark teal blue, respectively. The full version of this alignment is available in Fig. S4a. (C) The upper panel shows the schematic diagram of extant orthobornaviral genomic RNA, X/P mRNA, and miEBLX/P-4. Transcription signals (S2, T1, and T2) are indicated. The lower panel shows the nucleotide sequence alignment of extant orthobornaviral X/P transcripts and miEBLX/P-4. The light and dark teal blue letters indicate the start codon of X gene and the stop codon of P gene, respectively. The full version of this alignment is available in Fig. S5.

As described earlier, orthobornaviruses express mRNAs encoding N (1.2 kb), X and P (X/P mRNA, 0.7 kb), or N, X, and P (N/X/P mRNA, 1.9 kb) (Figs. 2A and S1). If miEBLN/X/P-1 is truly derived from N/X/P mRNA, there may be additional EBLs derived from N/X/P or X/P mRNA. To examine this possibility, we conducted a BLASTn search against bat genomes using the nt sequence of miEBLN/X/P-1 as a query. We obtained six hits from the genomes of *M. natalensis* and *M. schreibersii* (Fig. 3A and Table S1). While four hits were aligned in the entire N/X/P region of miEBLN/X/P-1 (Fig. 3A, red lines), interestingly, two other hits were aligned only to the putative X/P region (Fig. 3A, teal blue lines). Note that the N regions of four hits aligned to entire miEBLN/X/P-1 were detected as EBLNs in a previous study (Kawasaki et al. 2021).

We first assessed hits that aligned to the entire region of miEBLN/X/P-1. Four hits showed 1.9-kb alignment lengths and high sequence similarities (76–84 per cent nt identity) to miEBLN/X/P-1 (Table S1). The flanking regions of each pair of hit sequences were well aligned between *M. natalensis* and *M. schreibersii*, indicating that they are orthologous between these species (Table S8). We tentatively refer to these loci as miEBLN/X/P-2 and miEBLN/X/P-3. We also confirmed the presence of orthologs of these EBLN/X/Ps in the genome of *M. fuliginosus* by PCR and Sanger sequencing of genomic DNA isolated from *M. fuliginosus* (Fig. S2B and Table S8).

Next, we examined the presence of miEBLN/X/P-2 or miEBLN/X/P-3-empty loci by comparative genomics. We observed that the miEBLN/X/P-3-empty locus is present in genomes of non-miniopterid bats such as *T. brasiliensis* (Fig. S2B and Table S8). We did not obtain an alignment of the flanking region of miEBLN/X/P-2 with the other bat genomes and therefore could not identify its empty locus.

We then analyzed the nt sequences of miEBLN/X/P-2 and miEBLN/X/P-3. Similar to miEBLN/X/P-1, both EBLN/X/Ps contained transcription signal–like sequences, and poly-A stretches were observed downstream of T2-like sequences (Fig. S3). Furthermore, tandem duplication sequences, TGTTCA/[T/C]GTTCA and GAGGAACG/GAGGGATG, were also observed immediately upstream of S1 and downstream of T2/poly-A in miEBLN/X/P-2 and miEBLN/X/P-3, respectively (Fig. S3). These results suggest that miEBLN/X/P-2 and miEBLN/X/P-3 are also derived from ancient N/X/P transcripts and were integrated into the genome of a miniopterid bat ancestor through LINE-1 activity.

### EBLs derived from X/P-like transcripts exist in miniopterid bat genomes

Next, we assessed the hits that aligned to the 0.7-kb putative X/P region of miEBLN/X/P-1 (Fig. 3A, teal blue lines). Two hits from *M. natalensis* and *M. schreibersii* showed 0.8-kb alignment lengths and 75 per cent nt identity to the 0.7-kb X/P region (Table S1). The flanking regions of the hit sequences were well aligned, indicating that the loci are orthologous between these species (Figs. 3B and S4A; Table S8). We also found an orthologous locus in the genome of *M. fuliginosus* using PCR and Sanger sequencing (Figs. 3B and S4A; Table S8). Furthermore, we detected empty loci in genomes of non-miniopterid bats, i.e. genomes such as *Myotis lucifugus* (Figs. 3B and S4A; Table S8).

The orthobornaviral X/P mRNA contains the transcription signal sequences S2/T1 in 5ʹ-untranslated region (UTR) and T2 in 3ʹ-UTR (Fig. 3C). We observed that S2/T1- and T2-like sequences are present in the 0.7-kb insertion, which are located at positions similar to those in the extant X/P mRNA (Figs. 3C and S5). Poly-A stretches were also observed downstream of the T2-like sequences. Furthermore, the S2 and poly-A stretches were flanked by TSDs, CTTAAGGC/CTTAAGGC (Figs. 3C and S5). Taken together, these results indicated that the insertion may have been derived from an ancient X/P transcript and integrated into the genome of a miniopterid bat ancestor through LINE-1 activity. We tentatively refer to the insertion as the miEBLX/P-4.

### Preservation of intact ORFs in miEBLX/P-4 and characterization of the putatively encoded proteins

Most EBLs do not retain viral ORFs because of the accumulation of mutations after integration. Remarkably, miEBLX/P-4 retains intact X- and P-like ORFs in different reading frames, which is consistent with the extant orthobornaviral X/P mRNA (Fig. 3C). We named these ORFs as miEBLX-4 and miEBLP-4. Notably, miEBLP-4 in *M. natalensis* is disrupted by a premature stop codon.

Although there is very low sequence similarity between the amino acid sequences of these ORFs and extant orthobornaviral proteins, further analyses showed that miEBLX-4 and miEBLP-4 are intrinsically similar to extant orthobornaviral X and P proteins.

**Figure 4.**
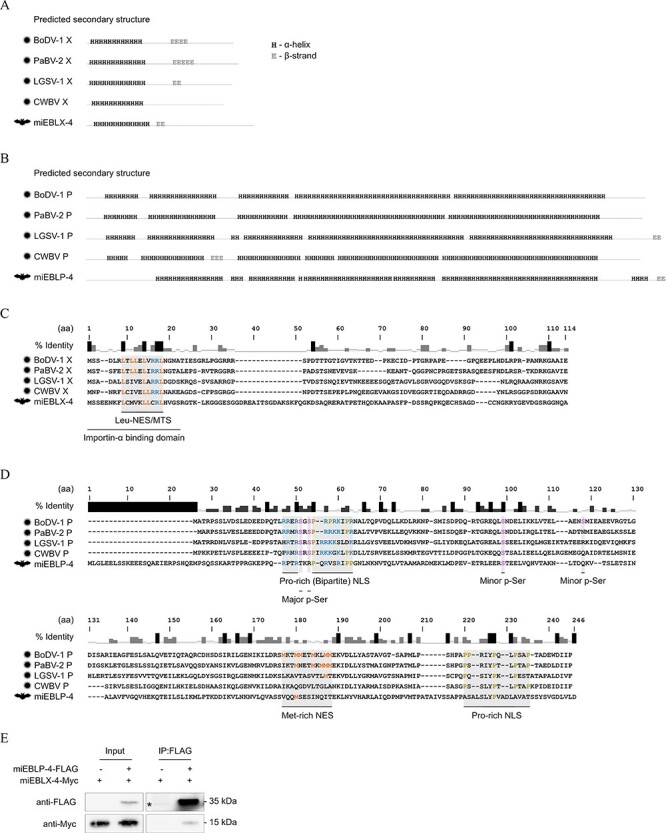
Conservation of properties between putative EBLX and EBLP and viral X and P proteins. The predicted secondary structure of extant orthobornaviral X and miEBLX-4 proteins (A) and extant orthobornaviral P and miEBLP-4 proteins (B). Representative viruses were selected from each clade of the orthobornavirus phylogenetic tree (Rubbenstroth et al. 2021) for the analyses. H, α-helix; E, β-strand. The data including amino acid residues are shown in Figs. S6 and S7. Amino acid sequence alignment of extant orthobornaviral X and miEBLX proteins (C) and extant orthobornaviral P and miEBLP-4 proteins (D). The shaded areas indicate the functional domains previously characterized in BoDV-1 X and P proteins. The conserved amino acid residues important for the functions of viral X or P protein are highlighted in colors. p-Ser, phosphorylated serine residue. (E) Co-immunoprecipitation of Myc-tagged miEBLX-4 with FLAG-tagged miEBLP-4. FLAG-tagged miEBLP-4 was immunoprecipitated and then analyzed by Western blotting using the indicated antibodies. *, non-specific band.

First, we examined the miEBLX-4 ORF. The ORF consists of 109 codons, which is longer than that of extant orthobornaviral X (86–90 codons). We performed a PHMMER search against the UniProt database using miEBLX-4 as a query, but no hit was detected. We then predicted the secondary structure and analyzed the hydrophobicity of the putative miEBLX-4 protein with respect to extant X proteins. Both extant X and miEBLX-4 were predicted to contain an alpha helix in the N-terminal region (Figs. 4A and S6A).

We also determined whether the functional domains are conserved between extant X and id sequence alignment showed that the N-terminal region is relatively well conserved and houses functional domains previously characterized in BoDV-1 X (Fig. 4C). This region includes the leucine-rich domain functioning both as a CRM1-dependent nuclear export signal (NES) (Ferre et al. 2016) and as a mitochondria-targeting signal (Poenisch et al. 2009) and is overlapped by the importin-alpha-binding domain (Wolff et al. 2002). The alignment also showed that leucine and arginine residues that are critical in the importin-alpha-binding domain (Fig. 4C; orange and blue, respectively) are conserved among extant X and miEBLX-4.

Next, we examined the miEBLP-4 ORF. The ORF consists of 233 codons, which is longer than those of extant orthobornaviral P (201–208 codons). A PHMMER search showed a significant similarity between amino acid sequences of miEBLP-4 and extant orthobornaviral P proteins (Table S2). We also detected a PHMMER hit in *Phyllostomus discolor*, which is identified as an EBLP in the later section (Diverse EBLX/Ps are found in vertebrate genomes). The predicted secondary structures and hydrophobicity pattern of miEBLP-4 protein were similar to those of the modern counterparts (Figs. 4B, S6B, and S7). Alignment of the amino acid sequences revealed that some of the functional domains previously characterized in BoDV-1 P may be shared between extant P and miEBLP-4 (Fig. 4D). Basic and proline residues important for the nuclear localization signal (NLS) activity (Fig. 4D, blue and yellow) (Shoya et al. 1998; Schwemmle et al. 1999) in the N-terminal NLS were prominent in both proteins.

We also explored the interaction between miEBLX-4 and miEBLP-4, which is a notable characteristic of the orthobornaviral X and P proteins (Fujino et al. 2012). We expressed Myc-tagged miEBLX-4 (miEBLX-4-Myc) and FLAG-tagged miEBLP-4 (miEBLP-4-FLAG) in 293T cells and then immunoprecipitated them using an anti-FLAG antibody. miEBLX-4-Myc was co-immunoprecipitated with miEBLP-4-FLAG, indicating the interaction between these proteins (Fig. 4E).

These results showed that miEBLX-4 and miEBLP-4 share similar intrinsic properties with the extant X and P proteins. Furthermore, together with the above-mentioned data, they strongly suggest that miEBLN/X/P-1–3 and miEBLX/P-4 are indeed derived from ancient viral N/X/P and X/P mRNAs, respectively.

### Diverse EBLX/Ps are found in vertebrate genomes

To further identify the presence of other EBLX/Ps, we conducted TFASTX and TFASTY searches against vertebrate genomes using the amino acid sequences of miEBLX-4 and miEBLP-4 as queries. TFASTX and TFASTY are sequence similarity search programs that allow frameshifts for alignment (Pearson et al. 1997). As a result, sixty-five unique hits (Tables S3–S6) were detected in the genomes of mammals (class Mammalia), reptiles (class Reptilia), amphibians (class Amphibia), and ray-finned fish (class Actinopterygii). The majority of these hits were detected in the bat genomes, including miEBLN/X/P-1, miEBLN/X/P-2, miEBLN/X/P-3, and miEBLX/P-4.

**Figure 5.**
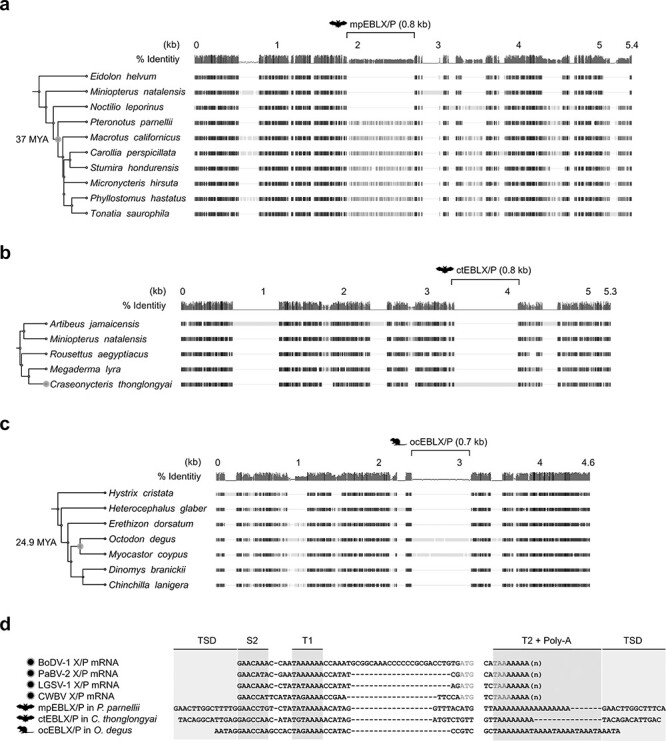
Identification of diverse EBLX/Ps in the vertebrate genomes. (A–C) Gene orthology analysis of each EBLX/P locus. Species phylogeny and deduced minimum integration age of each EBLX/P are indicated on the left based on TimeTree (Kumar et al. 2017). The full version of this alignment is available in Fig. S4. (D) nt sequence alignment of extant orthobornaviral X/P transcripts and EBLX/Ps. Black boxes show conserved residues. The thin gray lines indicate alignment gaps. Light and dark teal blue letters indicate the start codon of X gene and the stop codon of P gene, respectively. The full version of this alignment is available in Fig. S5.

Comparative genomic analyses strongly suggest that at least some of the TFASTX/Y hits are indeed derived from insertions of ancient viral X/P transcripts, as follows. TFASTX/Y hits in the bat families Mormoopidae and Phyllostomidae (mpEBLX/P) are orthologous, and their empty loci are present in other lineages of bats, such as *Noctilio leporinus* (Figs. 5A and S4B). For a TFASTX/Y hit in the lone bat species *Craseonycteris thonglongyai* (ctEBLX/P), we could not find its ortholog, but its empty sites exist in other bat species (Figs. 5B and S4C). We further identified orthologous TFASTX/Y hits in the rodent superfamily Octodontoidea and their empty loci in other lineages of rodents (ocEBLX/P; Figs. 5C and S4D). We also identified transcription signal–like sequences and TSDs within and flanking the above TFASTX/Y hits, respectively (Figs. 5D and S5). These results strongly suggested that at least some of the sequences detected by TFASTX and TFASTY are bona fide EBLX/Ps.

### Possible co-option of miEBLX/P-4 in miniopterid bats

Some EVEs, including EBLs, retain intact ORFs, which encode proteins that play roles in host-related functions (Horie 2017). As described earlier, miEBLX/P-4 contains intact ORFs corresponding to orthobornaviral X and P genes. Therefore, we analyzed miEBLX/P-4 in detail.

**Figure 6.**
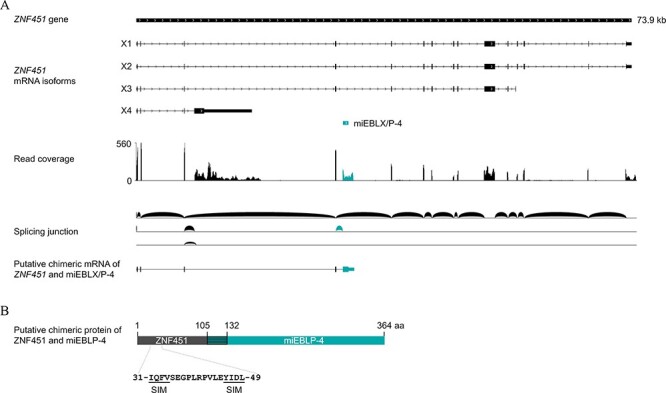
Alternative splicing generates *ZNF451*/*miEBLX/P-4* chimeric transcript. (A) Coverage and splicing junctions of mapped RNA reads from SuBK 12-08 cells (*M. schreibersii*) at the *ZNF451* locus where miEBLX/P-4 is located. The miEBLX/P-4 region, mapped read coverage graph on miEBLX/P-4, and splicing junction between ZNF451 and miEBLX/P-4 are shown in teal blue. (B) A schematic figure of putative chimeric ZNF451/miEBLP-4 protein. The shaded teal blue box indicates N-terminal extension of miEBLP-4 ORF within the miEBLX/P-4 region.

We analyzed the expression of miEBLX/P-4 by mRNA sequencing of *M. schreibersii*–derived SuBK12-08 cells. miEBLX/P-4 is located within the intron region of *ZNF451* gene. The read coverage of the miEBLX/P-4 locus was comparable to that of *ZNF451* (Fig. 6A). Interestingly, the mapping pattern revealed the existence of an alternative splicing site for *ZNF451* that could produce a chimeric transcript comprising ZNF451 exons 1–4 and the miEBLX/P-4 locus (Fig. 6A).

The putative chimeric transcript contains two ORFs consisting of more than 100 codons in different reading frames: a chimeric ORF of ZNF451 in frame with miEBLP-4 or that of the miEBLX-4 (Fig. 6B). The chimeric ORF potentially encodes a fusion protein comprising the N-terminal region of ZNF451, which contains a SUMO-interacting motif (SIM) domain, and miEBLP-4 (Fig. 6B).

These results suggest that miEBLX/P-4 has been co-opted by miniopterid bats as a chimeric gene with *ZNF451*.

## Discussion

We previously demonstrated that the X/P mRNA of a modern orthobornavirus can be reverse-transcribed and integrated into the host genome (Horie et al. 2010, 2013); however, no EBLX and only a few EBLPs have been reported (Kawasaki et al. 2021). We hypothesized that the technical limitations of the sequence similarity search hinder the detection of EBLs derived from the X and P genes. EBLs have been routinely searched using tBLASTn, but this can be an insensitive method for detecting small and rapidly evolving genes such as the X and P genes (Horie and Tomonaga 2019). In this study, our aim was to develop a novel strategy to detect these hidden EBLs. To this aim, we focused on the 1.9-kb read-through transcript of orthobornaviruses, consisting of a well-conserved N gene and small and rapidly evolving X and P genes (Poenisch et al. 2008), to detect hidden EBLX/Ps. We show a series of evidence implying that EBLN/X/Ps and EBLX/Ps are present in vertebrate genomes but are not detectable by BLAST searches (Figs 2 and 3). Interestingly, miEBLX/P-4 has maintained intact ORFs for more than 17 million years (Fig. 3) and is expressed as a fusion transcript with the cellular gene, *ZNF451*, which potentially encodes the ZNF451/miEBLP-4 fusion protein (Fig. 6). Therefore, this study revealed the presence of hidden EBLs, which could contribute to a deeper understanding of ancient bornaviruses and the co-evolution between bornaviruses and their hosts.

Here, we uncovered the potential co-option of miEBLX/P-4 by miniopterid bats, as indicated by the expression of the ZNF451/miEBLP-4 chimeric transcript, which potentially encodes a fusion protein consisting of the N-terminal region of ZNF451 and miEBLP-4 (Fig. 6). To the best of our knowledge, this is the first report of a non-retroviral EVE being expressed as a chimeric transcript with a known host gene. Although we could not confirm the expression of the ZNF451/miEBLP-4 fusion protein due to the lack of antibodies, our data suggest that miEBLX/P-4 has been co-opted by miniopterid bats. The BoDV-1 P protein has multiple functions, such as phosphorylation decoy to modulate the cellular signaling and induction of liquid–liquid phase separation (Unterstab et al. 2005; Schmid et al. 2007; Prat et al. 2009; Hirai, Tomonaga, and Horie 2021). Therefore, further studies are needed to understand the biological significance of miEBLX/P-4.

Our data strongly suggest that the genome organization and expression patterns of the N, X, and P genes have not substantially changed over the past millions of years. Here, we show that T1-read-through transcription occurred in ancient orthobornaviruses present at least 17.3 million years ago (MYA) (Fig. 2). Furthermore, the ORF positions of the X and P genes of ancient orthobornaviruses were highly similar to those of modern orthobornaviruses (Fig. 3). In addition, the sequences and order of transcription signals are highly conserved among ancient and modern orthobornaviruses (Figs 2 and 3). In BoDV-1, read-through transcription has been suggested to be involved in the regulation of viral gene expression (Poenisch et al. 2008). This hypothetical gene regulation mechanism has been maintained for more than 17.3 million years. Interestingly, our previous study showed that the order of S2-T1 is also conserved in some EBLNs in primates, which were estimated to be integrated into the host genomes more than 43 MYA (Belyi, Levine, and Skalka 2010; Horie et al. 2010; Katzourakis, Gifford, and Malik 2010). Thus, this gene regulatory mechanism may have been employed by orthobornaviruses earlier than 17.3 MYA.

The fundamental functions of viral X and P proteins may also be evolutionarily conserved between ancient and modern orthobornaviruses. We observed similarities in the intrinsic properties of extant orthobornaviral X and P proteins with miEBLX-4 and miEBLP-4 proteins despite their very low amino acid identities (Figs. 4, S6, and S7). Furthermore, the interaction between X and P may have been conserved in ancient orthobornaviruses (Fig. 4E). Therefore, although the evolutionary rates of X and P genes are high, the fundamental features and functions of X and P proteins may have been maintained for at least 17.3 million years.

For the EVE search, TFASTX/Y may be more suitable than tBLASTn, a conventional sequence similarity search for the detection of EVEs. After integration into the host genome, many EVEs have acquired random mutations, which caused frameshifts in the ORFs. Such frameshifted EVEs are fragmented and thus less detectable by tBLASTn. In contrast, TFASTX/Y takes frameshifts into account (Pearson et al. 1997) and can thus detect such fragmented EVEs more sensitively. We are currently conducting a benchmark analysis of tBLASTn and TFASTX/Y in detail, showing that some of the EBLX/Ps detected by TFASTX/Y cannot be detected by tBLASTn or other BLAST programs (data not shown). Therefore, TFASTX/Y can detect fragmented EVEs more sensitively than tBLASTn, which may contribute to identifying other hidden EVEs.

The EBLX/Ps identified in this study would be limited, and it is likely that there are still undetected EBLX/Ps in vertebrate genomes. In this study, the majority of EBLX/Ps were detected in bat and rodent genomes. Note that we failed to detect any EBLX/Ps in primate genomes, which contain numerous EBLs. Considering the number of EBLs in the genomes of primates (i.e. twenty-four orthobornaviral EBLs were detected in the human genome (Kawasaki et al. 2021)), it is plausible that hidden EBLX/Ps exist in primates that are undetectable because of the inherent low amino acid similarities in X and P.

We may be able to detect such hidden EBLX/Ps by identifying other EBLN/X/P integrations. However, searching for other EBLN/X/Ps by manual curation is time-consuming. Thus, the development of an efficient searching method to identify approximately 1.9-kb insertions with EBLN would facilitate further exploration of EBLX/Ps. This is probably not limited to bornaviruses; there may be many hidden EVEs that are derived from small and/or rapidly evolving viral genes. Read-through transcription has also been observed for other mononegaviruses (Banerjee et al. 1991). Our search strategy can be applied to the detection of such EVEs. Additionally, a previous study identified virus-like insertions using a machine learning–based method, but the origins of insertions are still unclear (Kojima et al. 2021). Our approach might also contribute to understanding the origins of those sequences.

Taken together, we identified EBLs that were undetectable by conventional BLAST searches. Our findings provide novel insights into ancient bornaviruses and co-evolution between bornaviruses and their hosts. Furthermore, our search strategy will contribute to paleovirological research.

## Materials and methods

### Sanger sequencing of EBLs and flanking loci in *M. fuliginosus*

Genomic DNA was obtained from YubFKT1 (kidney epithelial cell line) (Maeda et al. 2008) in a previous study (Mukai et al. 2022). miEBLN/X/P-1, miEBLN/X/P-2, miEBLN/X/P-3, and miEBLX/P-4 and corresponding flanking loci in *M. fuliginosus* were amplified by PCR using the extracted DNA and primers (Table S7) with Phusion Hot Start II High-Fidelity PCR Master Mix (Thermo Fisher Scientific). The PCR conditions are available upon request. The amplicons were purified using the innuPREP PCRpure Lite Kit (Analytik Jena) and sequenced by the Sanger dideoxy method at Eurofins Genomics. The primer sequences are available in Table S6. The obtained sequences were deposited to DNA Data Bank of Japan (DDBJ) (accession numbers LC708263–LC708266).

### Cell lines

SuBK12-08 cells (Maruyama et al. 2014) and human embryonic kidney 293T cells were cultured in low-glucose Dulbecco’s modified Eagle medium (Nacalai Tesque) supplemented with 10 per cent fetal bovine serum (Sigma-Aldrich) and 100 μg/ml penicillin-streptomycin (Nacalai Tesque). The cells were maintained at 37°C in a humidified chamber containing 5 per cent CO_2_.

### Construction of miEBLX-4- and miEBLP-4-expressing plasmids

Expression vectors with C-terminal FLAG or Myc tag were first constructed. pcDNA3 was digested with EcoRI and XhoI, which was assembled with oligo DNA containing the Myc or FLAG tag sequence using NEBuilder HiFi DNA Assembly Master Mix (New England Biolabs). The resultant plasmids were named pcDNA3-C-Myc or pcDNA3-C-FLAG. Then, miEBLX-4 and miEBLP-4 were amplified by PCR using the genomic DNA extracted from YubFKT1 with Phusion Hot Start II High-Fidelity PCR Master Mix, which were subsequently assembled with pcDNA3-C-Myc or pcDNA3-C-FLAG linearized with KpnI and EcoRI using NEBuilder. The oligo DNA sequences are available in Table S7.

### Co-immunoprecipitation of miEBLX-4 with miEBLP-4

pcDNA3-miEBLX-4-Myc and pcDNA3-miEBLP-4-FLAG plasmids (5 μg) were transfected into 293T cells on a 10-cm dish using 20 μl of Avalanche-Everyday Transfection Reagent (EZ Biosystems) with Opti-MEM (Thermo Fisher Scientific). One-day post-transfection, the cells were washed twice with ice-cold PBS and lysed using 500 μl of immunoprecipitation (IP) buffer (150 mM NaCl, 20 mM Tris-HCl pH 7.4, 1 mM EDTA , 1 per cent Triton X-100, 1× protease inhibitor cocktail [Nacalai Tesque]) for 30 min at 4°C with rotation. In parallel, 500 μg of SureBeads Protein G magnetic beads (Bio-Rad) were incubated with 3 μg of mouse monoclonal anti-DDDDK antibody FLA-1 (M185-3L; MBL) in IP buffer for 1 h at 4°C with rotation. The lysates were then centrifuged at 12,000 × g for 10 min at 4°C, and then the supernatant was immunoprecipitated with the antibody–bead complex in IP buffer for 2 h in 4°C with rotation. After incubation, the beads were washed thrice with ice-cold IP buffer and then transferred into new microtubes. The proteins were finally eluted from the beads by boiling in SDS sample buffer for 5 min at 95°C.

### Western blotting

Protein samples were separated in 12 per cent polyacrylamide gel in Tris-glycine-SDS buffer, which were transferred to the PVDF membrane (MERCK) at 10 V for 1 h using the Trans-Blot Turbo Transfer System (Bio-Rad). Then, the membrane was blocked with 5 per cent skim milk in 0.1 per cent Tween 20 in PBS for 1 h at room temperature (RT). The membrane was incubated with mouse monoclonal anti-Myc My3 (MBL; 1:5000) or anti-DDDDK FLA-1 antibody (MBL; 1:2000) diluted in 5 per cent skim milk for 1 h at RT, washed, and then incubated with horseradish peroxidase–conjugated donkey polyclonal anti-mouse IgG (715-035-150, Jackson ImmunoResearch; 1:2000) antibody diluted in 5 per cent skim milk for 45 min at RT. The membrane was washed again, after which chemiluminescence reaction was conducted using Chemi-Lumi One (Nacalai Tesque) and was subsequently viewed using Amersham Imager 680 (GE Healthcare).

### BLAST search against bat genomes to detect EBLN/X/Ps and EBLX/Ps

BLASTn (Camacho et al. 2009) search was conducted against the Whole Genome Shotgun (WGS) database (Coordinators 2018) of bat (Chiroptera, taxid: 9397) using the nt sequence of miEBLN/X/P-1 in *M. natalensis* as a query on the BLAST web server (https://blast.ncbi.nlm.nih.gov/Blast.cgi). Changes applied to the default parameters are as follows: *E*-value threshold = 10^–10^, word size = 7, and no filter for low-complexity regions.

### HMMER search using miEBLX-4 and miEBLX-P

PHMMER search was performed on the HMMER web server (Finn, Clements, and Eddy 2011) with the default setting using the putative amino acid sequences of miEBLX-4 and miEBLP-4 as queries. The obtained hits were sorted by domain-independent *E*-value.

### TFASTX/Y search against vertebrate genomes to detect EBLX/Ps

Vertebrate genomes were downloaded using NCBI datasets and were used as the database for TFASTX/Y searches. TFASTX/Y searches, included in the FASTA36 package (Pearson 2016), were performed against each species of the vertebrate genomes using the putative amino acid sequences miEBLX-4 and miEBLP-4 as queries with the default parameters, except for the ktup value option of 1. The obtained hits with *E*-values less than 10^–4^ were extracted, which were used for the downstream analyses.

### Identification of orthologous and syntenic empty loci

BLASTn search was conducted against the WGS database of bats (Chiroptera, taxid: 9397) or rodents (Rodentia, taxid: 9989) using the nt sequences of EBL and its flanking regions as queries on the BLAST web server. Changes applied to the default parameters are as follows: *E*-value = 10^–20^ and word size = 11. The BLAST hits that comprised genomic sequences from different species were retrieved and then aligned by MAFFT using the E-INS-i algorithm (Katoh and Standley 2013) in Geneious version 11.1.5. The nt accessions and region of EBLs used for search queries are available in Table S8.

### Annotation of EBLs and TSDs

TSD sequences were determined based on the alignment made in ‘Identification of orthologous and syntenic empty loci’. To identify transcription signal–like sequences, the nt sequences of EBLs and extant orthobornaviral N/X/P or X/P transcripts were aligned by MAFFT using the E-INS-i algorithm. The transcription signal–like sequences and poly-A stretches were determined according to the resultant alignment.

To identify conserved functional domains of the miEBLX-4 and miEBLP-4 proteins, the amino acid sequences of miEBLX-4p and miEBLP-4p in *M. fuliginosus* were aligned with extant orthobornaviral X and P proteins, respectively, by MAFFT using the E-INS-i algorithm. Based on the alignments, the conserved domains were manually analyzed.

The nt accession of extant orthobornaviruses used in the analysis is available in Table S9.

### mRNA sequencing analysis

Total RNA was extracted from SuBK12-08 cells using NucleoSpin RNA plus (MACHEREY-NAGEL) according to the manual. Sequencing library was constructed using the TruSeq Stranded mRNA Library Prep Kit (Illumina). The libraries were sequenced by NextSeq 500 using NextSeq 500/550 Mid Output Kit v2.5 (300 cycles).

The obtained reads were preprocessed using fastp 0.23.2 (Chen et al. 2018) with the default setting, which were mapped to the reference genome of *M. natalensis* (GCF_001595765.1) using HISAT2 version 2.2.1 (Kim et al. 2019) with the default setting. The mapped reads and splicing junctions were visualized using Integrated Genomics Viewer 2.13.0 (Robinson et al. 2011).

The RNA-sequencing data were deposited to DDBJ Sequence Read Archive (DRA) under the accession number DRR403400.

### Pairwise similarity analysis

Amino acid sequences of the viral proteins of extant orthobornaviruses (Table S8) were analyzed by the MAFFT alignment tool using Sequence Demarcation Tool software version 1.2 (Muhire, Varsani, and Martin 2014). The minimum value for the percent identity scale was set to 0.1. The resulting matrices were set to three-color mode and exported.

### In silico characterization of putative EBLX and EBLP proteins

To predict the protein secondary structure, amino acid sequences of miEBLX-4 and miEBLP-4 in *M. fuliginosus*, and extant orthobornaviral X and P proteins, were analyzed using JPred version 4 (Drozdetskiy et al. 2015).

To determine the protein hydropathy, the same amino acid sequences were submitted to ProtScale (Walker 2005) in ExPASy Server, using the amino acid scale developed by Kyte and Doolittle. The window sizes were set to 9 and 15 for X and P proteins, respectively.

## Supplementary Material

vead038_Supp

## Data Availability

The data were available at DDBJ and DRA under the accession numbers LC708263–LC708266 and DRR403400, respectively.
