# Supplementary material for: The hidden diversity of ancient bornaviral sequences from X and P genes in vertebrate genomes
Source: Virus Evol. 2023 Jun 3;9(1):vead038. doi: 10.1093/ve/vead038 (PMC10288550; doi:10.1093/ve/vead038)
Supplement: vead038_Supp [file vead038_supp.zip › Tables_supp.pdf]

Table S1. BLASTn hits using miEBLN/X/P-1 in *Miniopterus natalensis* as query.

Subject accession	Subject species	% Identity	Alignment length	Query start*	Query end*	Subject start	Subject end	*E* value	Bit score	Definition
LDJU01000053.1	*Miniopterus natalensis*	100.00	1887	1	1887	443709	441823	0	3404	miEBLN/X/P-1
PVJG01001939.1	*Miniopterus schreibersii*	98.26	1897	1	1887	114525	112630	0	3265	miEBLN/X/P-1
PVJG01000137.1	*Miniopterus schreibersii*	84.50	1910	1	1867	102880	104779	0	2086	miEBLN/X/P-2
LDJU01000641.1	*Miniopterus natalensis*	84.40	1910	1	1867	199193	197294	0	2077	miEBLN/X/P-2
PVJG01014896.1	*Miniopterus schreibersii*	76.43	1939	1	1872	30174	28318	0	1396	miEBLN/X/P-3
LDJU01000514.1	*Miniopterus natalensis*	76.48	1939	1	1872	756607	758473	0	1396	miEBLN/X/P-3
PVJG01006939.1	*Miniopterus schreibersii*	75.70	790	1147	1872	45379	44596	3.86E-158	569	miEBLX/P-4
LDJU01000157.1	*Miniopterus natalensis*	75.35	787	1147	1872	192899	193680	8.50E-154	555	miEBLX/P-4
LDJU01000096.1	*Miniopterus natalensis*	90.74	216	1682	1887	4262053	4262268	2.98E-77	300	-
PVJG01004446.1	*Miniopterus schreibersii*	90.28	216	1682	1887	74472	74257	1.27E-75	296	-
PVJL010000181.1	*Megarderma lyra*	70.58	435	423	849	430769	430354	9.13E-46	196	-
AWHB01421187.1	*Megarderma lyra*	70.35	435	423	849	1000	1415	1.11E-44	192	-
VMDN01009446.1	*Rhinolophus ferrumequinum*	65.71	627	208	831	17620	18236	1.76E-29	142	-
RXPC01000085.1	*Rhinolophus ferrumequinum*	65.71	627	208	831	661690	661074	1.76E-29	142	-
JACAGC010000009.1	*Rhinolophus ferrumequinum*	65.71	627	208	831	574429	573813	1.76E-29	142	-
AWHA01050524.1	*Rhinolophus ferrumequinum*	65.71	627	208	831	4732	5348	1.76E-29	142	-
PVJD01006365.1	*Mormoops blainvillei*	65.90	610	445	1044	71722	72322	6.16E-29	140	-
PVJD01006365.1	*Mormoops blainvillei*	68.16	245	121	359	71150	71394	1.45E-11	83.3	-
RXPD01008596.1	*Rhinolophus ferrumequinum*	65.55	627	208	831	12939	13555	2.15E-28	138	-
RXPD01003538.1	*Rhinolophus ferrumequinum*	65.55	627	208	831	247206	247822	2.15E-28	138	-
VMDR010000046.1	*Macrotus californicus*	63.70	909	178	1073	277198	278094	1.36E-24	125	-
AWGZ01398077.1	*Pteronotus parnellii*	67.84	342	445	782	28	361	2.45E-21	114	-
PVJD01002030.1	*Mormoops blainvillei*	63.28	1024	157	1166	120486	121491	8.56E-21	113	-
JAHKBD010000144.1	*Phyllostomus hastatus*	63.90	867	157	1001	27586564	27585720	2.99E-20	112	-
JAIWKQ010000146.1	*Pteronotus parnellii*	63.78	693	125	782	3935098	3934421	1.27E-18	105	-
PEHR01060085.1	*Desmodus rotundus*	70.51	217	126	342	13330	13114	4.43E-18	104	-
JAKFFZ010000003.1	*Desmodus rotundus*	70.51	217	126	342	130229422	130229206	4.43E-18	104	-
PVJG01000099.1	*Miniopterus schreibersii*	81.55	103	1785	1879	269931	269829	1.88E-16	99.6	-
LDJU01000026.1	*Miniopterus natalensis*	81.55	103	1785	1879	2093767	2093869	1.88E-16	99.6	-
JAKFGA010000007.1	*Desmodus rotundus*	70.05	217	126	342	130145647	130145431	1.88E-16	99.6	-
PVIA01003908.1	*Tonatia saurophila*	64.10	649	424	1067	56279	56917	1.88E-16	98.7	-
PVJI01011652.1	*Micronycteris hirsuta*	63.80	721	187	896	50597	49886	6.58E-16	97.8	-
AWGZ01165285.1	*Pteronotus parnellii*	62.88	862	157	1000	1909	1062	3.41E-13	87.8	-
RXPB02005429.1	*Phyllostomus discolor*	64.12	563	446	1001	50127	49577	1.19E-12	86.9	-
JAIWKQ010000280.1	*Pteronotus parnellii*	62.65	929	157	1067	9632211	9631297	1.19E-12	86.9	-
JABVXQ010000005.1	*Phyllostomus discolor*	64.12	563	446	1001	47764028	47764578	1.19E-12	86.9	-
AWGZ01125890.1	*Pteronotus parnellii*	69.09	220	125	339	293	74	1.19E-12	86.9	-
RXPA02000005.1	*Phyllostomus discolor*	64.30	563	446	1001	127497467	127496919	4.15E-12	84.2	-
PVKU01009917.1	*Anoura caudifer*	64.56	474	424	896	38072	37606	1.45E-11	83.3	-
PVKR01001528.1	*Artibeus jamaicensis*	67.42	264	427	689	92697	92438	5.06E-11	80.6	-
JAIVGF010000003.1	*Artibeus jamaicensis*	67.42	264	427	689	20906667	20906926	5.06E-11	80.6	-

*Query: miEBLN/X/P-1 in *Miniopterus natalensis* (LDJU01000053.1:443709-441823)

Tables S2. PHMMER search hits using miEBLP-4 in *Miniopterus fuliginosus* as query.

Target accession	Target species	Target Length	E value	Score	Bias	Domain index	Domain count	Conditional E value	Domain independent E value	Domain bit score	Domain bias	Query alignment start*	Query alignment end*	Target alignment start	Target alignment end	Target envelope start	Target envelope end	Accuracy	Description	Mapped PDB(s)	Number of Identical Sequences
A0A834EQ99_9CHIR	Phyllostomus discolor	145	8.90E-21	88.1	0.2	1	1	5.30E-27	9.80E-21	87.94	0.21	79	227	1	144	1	145	0.96	Uncharacterized protein		0
G3KJK0_9MONO	Aquatic bird bornavirus 1	193	9.20E-06	39.1	4.3	1	1	6.00E-12	1.10E-05	38.83	4.26	40	214	15	189	1	192	0.75	Phosphoprotein (Fragment)		0
A0A0M4AXZ0_9MONO	Aquatic bird bornavirus 1	202	1.10E-05	38.9	3.8	1	1	6.80E-12	1.20E-05	38.65	3.79	40	214	15	187	1	200	0.73	Phosphoprotein		0
A0A0S0GLH4_9MONO	Aquatic bird bornavirus 1	202	1.40E-05	38.5	3.3	1	1	8.80E-12	1.60E-05	38.28	3.26	40	214	15	187	1	200	0.73	Phospoprotein		0
A0A0S0GL96_9MONO	Aquatic bird bornavirus 1	202	1.50E-05	38.4	3.7	1	1	9.30E-12	1.70E-05	38.20	3.71	40	206	15	181	1	200	0.74	Phosphoprotein		0
H9BW78_9MONO	Canary bornavirus 3	202	1.40E-05	38.5	2.9	1	1	9.80E-12	1.80E-05	38.12	2.85	27	203	2	184	1	199	0.75	Putative phosphoprotein		0
A0A0S0FAL8_9MONO	Aquatic bird bornavirus 1	202	1.60E-05	38.3	3.7	1	1	1.00E-11	1.90E-05	38.05	3.66	40	205	15	180	1	200	0.74	Phospoprotein		0
A0A1B3ITA5_9MONO	Aquatic bird bornavirus 1	202	1.90E-05	38	3.2	1	1	1.20E-11	2.20E-05	37.81	3.19	40	206	15	181	1	200	0.74	Phosphoprotein		0
M9V366_9MONO	Canary bornavirus 3	202	3.20E-05	37.3	2.8	1	1	2.30E-11	4.20E-05	36.93	2.77	36	203	11	184	1	199	0.74	Phosphoprotein		0
A0A1B3IT87_9MONO	Aquatic bird bornavirus 1	202	3.50E-05	37.2	3.3	1	1	2.30E-11	4.20E-05	36.94	3.32	40	214	15	187	1	200	0.73	Phosphoprotein		0
M9V5K5_9MONO	Canary bornavirus 1	202	3.80E-05	37.1	3.9	1	1	2.70E-11	5.00E-05	36.68	3.91	41	206	16	187	5	199	0.78	Phosphoprotein		0
M9V340_9MONO	Canary bornavirus 1	202	4.50E-05	36.8	4	1	1	3.20E-11	5.80E-05	36.46	4	41	206	16	187	5	199	0.77	Phosphoprotein		0
A0A1B3ITA7_9MONO	Canary bornavirus 1	202	4.80E-05	36.7	4.2	1	1	3.40E-11	6.20E-05	36.36	4.23	41	206	16	187	5	199	0.78	Phosphoprotein		0
M9V338_9MONO	Canary bornavirus 1	202	5.00E-05	36.7	4.2	1	1	3.50E-11	6.50E-05	36.31	4.19	41	206	16	187	5	199	0.77	Phosphoprotein		0
V9XQU2_9MONO	Estrildid finch bornavirus 1	202	5.50E-05	36.5	4.5	1	1	3.50E-11	6.50E-05	36.31	4.46	40	206	15	183	1	199	0.77	Phosphoprotein		0
M9V5J7_9MONO	Canary bornavirus 1	202	6.00E-05	36.4	4.2	1	1	4.30E-11	7.90E-05	36.03	4.24	41	206	16	187	5	199	0.77	Phosphoprotein		0
I3RJF9_9MONO	Aquatic bird bornavirus 1	178	1.00E-04	35.7	4.3	1	1	6.40E-11	1.20E-04	35.48	4.33	40	196	15	177	1	178	0.77	Phosphoprotein (Fragment)		1
M9V5C4_9MONO	Canary bornavirus 1	202	1.20E-04	35.4	3.7	1	1	8.60E-11	0.00016	35.05	3.67	41	206	16	187	5	199	0.77	Phosphoprotein		0
C5I0W1_9MONO	Parrot bornavirus 2	201	0.00015	35.1	4.6	1	1	1.00E-10	0.00019	34.77	4.59	42	199	16	179	3	199	0.78	P protein		0
A0A649YJ81_9MONO	Parrot bornavirus 2	201	0.00016	35	4.6	1	1	1.10E-10	0.00021	34.66	4.57	42	199	16	179	3	199	0.79	Phosphoprotein		0
A0A076NB66_9MONO	Aquatic bird bornavirus 2	202	0.00021	34.6	3	1	1	1.50E-10	0.00027	34.29	3.01	39	206	14	183	1	199	0.73	Phosphoprotein		0
M9V362_9MONO	Canary bornavirus 2	202	0.00022	34.6	2.8	1	1	1.50E-10	0.00027	34.27	2.82	40	207	15	188	1	198	0.75	Phosphoprotein		0
M9V358_9MONO	Canary bornavirus 2	202	0.00024	34.5	2.8	1	1	1.6E-10	0.00029	34.20	2.75	40	202	15	183	1	200	0.74	Phosphoprotein		0
M9V5K9_9MONO	Canary bornavirus 2	202	0.00031	34.1	2.7	1	1	2.00E-10	0.00037	33.83	2.68	40	202	15	183	1	200	0.74	Phosphoprotein		0
E7D0B4_9MONO	Parrot bornavirus 2	201	0.00031	34.1	4.7	1	1	2.20E-10	0.0004	33.73	4.66	42	199	16	179	3	199	0.78	P protein		0
B4ZYJ7_9MONO	Parrot bornavirus 2	201	0.00033	34	4.8	1	1	2.40E-10	0.00043	33.62	4.77	42	199	16	179	3	199	0.78	Phosphoprotein		0
A0A060L6H9_9MONO	Parrot bornavirus 2	201	0.00038	33.8	4.5	1	1	2.70E-10	0.00049	33.45	4.48	42	199	16	179	3	199	0.78	Phosphoprotein		0
I3RJF6_9MONO	Aquatic bird bornavirus 1	178	0.00045	33.6	3.7	1	1	2.80E-10	0.00052	33.36	3.73	40	196	15	177	1	178	0.77	Phosphoprotein (Fragment)		0
F6K7D7_9MONO	Parrot bornavirus 2	201	0.00042	33.7	4.7	1	1	2.90E-10	0.00054	33.30	4.65	42	199	16	179	3	199	0.78	Phosphoprotein		0
A0A060LD59_9MONO	Parrot bornavirus 4	201	0.00046	33.5	4	1	1	3.00E-10	0.00056	33.26	4.03	42	199	16	179	3	201	0.75	Phosphoprotein		0
A0A650E6R1_9MONO	Parrot bornavirus 4	201	0.00044	33.6	2.5	1	1	3.10E-10	0.00057	33.23	2.5	42	215	16	193	3	198	0.76	Phosphoprotein		0
G9F7M4_9MONO	Parrot bornavirus 4	201	0.0005	33.4	4.3	1	1	3.30E-10	0.00061	33.14	4.27	42	207	16	187	3	201	0.76	P protein		0
F6JSK3_9MONO	Parrot bornavirus 4	201	0.00053	33.3	4.2	1	1	3.50E-10	0.00064	33.07	4.21	42	199	16	179	3	201	0.75	P		0
B8X6M0_9MONO	Parrot bornavirus 3	201	0.00054	33.3	4.4	1	1	3.70E-10	0.00068	32.98	4.42	44	201	20	181	2	200	0.77	P		0
G9F7N0_9MONO	Parrot bornavirus 4	201	0.00083	32.7	4	1	1	5.40E-10	0.00099	32.45	3.98	42	199	16	179	3	201	0.75	Phosphoprotein		0

F6JSJ7_9MONO	Parrot bornavirus 1	201	0.0008	32.7	4.6	1	1	5.80E-10	0.0011	32.35	4.56	42	200	16	180	3	199	0.77	P protein	0
A0A1B3ITH9_9MONO	Parrot bornavirus 4	201	0.001	32.4	4.2	1	1	6.50E-10	0.0012	32.18	4.15	43	199	17	179	3	201	0.76	Phosphoprotein	0
I6YQ72_9MONO	Parrot bornavirus 4	201	0.0015	31.9	4.5	1	1	9.80E-10	0.0018	31.60	4.46	42	201	16	181	3	201	0.75	Phosphoprotein	0
I6ZY70_9MONO	Parrot bornavirus 1	201	0.0014	31.9	4.9	1	1	1.00E-09	0.0019	31.54	4.88	42	200	16	180	3	199	0.76	Phosphoprotein	0
A0A0H5BWK0_9MONO	Variegated squirrel bornavirus 1	202	0.0018	31.6	4.4	1	1	1.20E-09	0.0022	31.34	4.44	40	197	15	178	1	200	0.74	P	0
I6YQ82_9MONO	Parrot bornavirus 7	201	0.002	31.5	3.7	1	1	1.40E-09	0.0025	31.11	3.66	42	199	16	179	3	199	0.77	Phosphoprotein	0
A0A1D3JBL2_9MONO	Variegated squirrel bornavirus 1	202	0.0022	31.3	4.4	1	1	1.40E-09	0.0026	31.07	4.44	40	197	15	178	1	200	0.73	Phosphoprotein	0
A0A649YJ72_9MONO	Parrot bornavirus 7	201	0.0037	30.6	3.7	1	1	2.4E-09	0.0044	30.32	3.67	45	199	21	179	3	201	0.75	Phosphoprotein	0
A0A1D3JBE1_9MONO	Variegated squirrel bornavirus 1	202	0.0043	30.4	3.6	1	1	3.00E-09	0.0054	30.04	3.59	45	197	22	178	3	200	0.75	p23	0
Q5GLC9_BDV1	Borna disease virus 1	201	0.0056	30	2.2	1	1	4.10E-09	0.0074	29.59	2.23	40	196	15	177	3	199	0.73	P protein	0
A0A6M3I0H2_9MONO	Mammalian 2 orthobornavirus	202	0.0081	29.5	4	1	1	5.70E-09	0.01	29.10	4.01	44	197	19	178	2	200	0.71	Phosphoprotein	0
Q64GK8_BDV1	Borna disease virus 1	202	0.0096	29.2	1.8	1	1	7.20E-09	0.013	28.77	1.76	40	208	15	189	5	193	0.75	p24	0
A0A2T0Q1E9_9BURK	Paraburkholderia sp. BL25I1N1	1498	4.50E-08	46.6	24.8	2	4	1.90E-05	34	17.64	0.28	110	222	719	834	711	843	0.81	Trimeric autotransporter adhesin (Fragment)	0
A0A4R8K685_9BURK	Paraburkholderia sp. BL6665CI2N2	2692	1.60E-19	84	42	5	7	8.80E-05	160	15.43	0.24	111	212	1560	1658	1552	1677	0.75	Trimeric autotransporter adhesin	0
A0A2T0QFP4_9BURK	Paraburkholderia sp. BL25I1N1	1071	2.50E-06	40.9	13.3	2	3	0.00029	530	13.75	0.27	144	229	216	308	186	311	0.76	Trimeric autotransporter adhesin (Fragment)	0
A0A2T0Q1E9_9BURK	Paraburkholderia sp. BL25I1N1	1498	4.50E-08	46.6	24.8	1	4	0.00036	660	13.44	0.15	124	212	287	378	266	395	0.75	Trimeric autotransporter adhesin (Fragment)	0
A0A2T0QFP4_9BURK	Paraburkholderia sp. BL25I1N1	1071	2.50E-06	40.9	13.3	3	3	0.00039	720	13.32	0.1	122	228	414	527	351	530	0.79	Trimeric autotransporter adhesin (Fragment)	0
A0A2T0QFP4_9BURK	Paraburkholderia sp. BL25I1N1	1071	2.50E-06	40.9	13.3	1	3	0.0004	740	13.28	0.38	110	212	43	143	36	160	0.76	Trimeric autotransporter adhesin (Fragment)	0
A0A3D9MBL0_9BURK	Paraburkholderia sp. BL27I4N3	3204	8.10E-17	75.2	76.3	9	10	0.00048	880	13.03	0.24	110	229	2312	2440	2304	2443	0.79	Trimeric autotransporter adhesin	0
A0A3E0CN26_9BURK	Paraburkholderia sp. BL6669N9	3173	1.00E-23	97.7	50.6	8	9	0.0005	920	12.96	0.42	110	229	2289	2410	2282	2413	0.73	Trimeric autotransporter adhesin	0
A0A495GKR6_9BURK	Paraburkholderia sp. BL17N1	1417	5.10E-11	56.2	15.9	3	5	0.0005	930	12.96	0.11	126	214	874	963	851	980	0.79	Trimeric autotransporter adhesin	0
A0A4R1UP80_9BURK	Paraburkholderia sp. GV060	3796	5.70E-20	85.5	85.2	11	11	0.00053	970	12.89	0.16	111	223	2815	2923	2807	2934	0.76	Trimeric autotransporter adhesin	0
A0A4R8K685_9BURK	Paraburkholderia sp. BL6665CI2N2	2692	1.60E-19	84	42	6	7	0.00055	1000	12.83	0.31	111	223	1839	1948	1831	1955	0.76	Trimeric autotransporter adhesin	0
A0A1H5DYA5_9BURK	Burkholderia sp. WP9	4846	1.30E-15	71.2	66.2	9	9	0.00056	1000	12.82	0.31	145	212	3787	3857	3757	3877	0.78	Head domain of trimeric autotransporter adhesin	0
A0A2T0Q1E9_9BURK	Paraburkholderia sp. BL25I1N1	1498	4.50E-08	46.6	24.8	3	4	0.00055	1000	12.84	0.19	126	222	1102	1199	1078	1208	0.78	Trimeric autotransporter adhesin (Fragment)	0
A0A495GKR6_9BURK	Paraburkholderia sp. BL17N1	1417	5.10E-11	56.2	15.9	2	5	0.00067	1200	12.55	0.12	126	214	511	600	486	617	0.79	Trimeric autotransporter adhesin	0
B2SYM9_PARPJ	Paraburkholderia phytofirmans PsJN	3635	2.70E-31	122.4	69.8	3	14	0.00069	1300	12.53	0.23	142	212	1256	1328	1228	1348	0.77	YadA domain protein	0
I2IG26_9BURK	Burkholderia sp. Ch1-1	2342	2.10E-09	51	16.3	5	5	0.00068	1300	12.53	0.13	142	212	1980	2053	1949	2071	0.76	Hemagglutinin-like protein (Fragment)	0
A0A3D9MBL0_9BURK	Paraburkholderia sp. BL27I4N3	3204	8.10E-17	75.2	76.3	6	10	0.00091	1700	12.12	0.32	111	222	1439	1547	1431	1556	0.74	Trimeric autotransporter adhesin	0
A0A3E0CN26_9BURK	Paraburkholderia sp. BL6669N3	3173	1.00E-23	97.7	50.6	2	9	0.00097	1800	12.03	0.27	110	212	1395	1501	1387	1518	0.82	Trimeric autotransporter adhesin	0
A0A3E0CN26_9BURK	Paraburkholderia sp. BL6669N5	3173	1.00E-23	97.7	50.6	4	9	0.00097	1800	12.03	0.27	110	212	1695	1801	1687	1818	0.82	Trimeric autotransporter adhesin	0
A0A3E0CN26_9BURK	Paraburkholderia sp. BL6669N7	3173	1.00E-23	97.7	50.6	6	9	0.00097	1800	12.03	0.27	110	212	1995	2101	1987	2118	0.82	Trimeric autotransporter adhesin	0
B2SYM9_PARPJ	Paraburkholderia phytofirmans PsJN	3635	2.70E-31	122.4	69.8	13	14	0.001	1900	11.95	0.16	143	212	2808	2880	2780	2902	0.75	YadA domain protein	0
B2SYM9_PARPJ	Paraburkholderia phytofirmans PsJN	3635	2.70E-31	122.4	69.8	7	14	0.0011	2000	11.84	0.09	142	212	1822	1895	1790	1915	0.72	YadA domain protein	0
A0A5Q4Z2J2_9BURK	Paraburkholderia sp. Msb3	3475	9.10E-21	88.1	47.5	3	6	0.0011	2000	11.90	0.13	142	211	932	1004	901	1023	0.76	Putative membrane-anchored cell surface protein, haemagluttinin	0
I2IG26_9BURK	Burkholderia sp. Ch1-1	2342	2.10E-09	51	16.3	3	5	0.0011	2100	11.81	0.16	123	212	1147	1239	1128	1258	0.75	Hemagglutinin-like protein (Fragment)	0
A0A495GKR6_9BURK	Paraburkholderia sp. BL17N1	1417	5.10E-11	56.2	15.9	5	5	0.0012	2200	11.76	0.17	124	212	1163	1254	1144	1271	0.75	Trimeric autotransporter adhesin	0

B2SYM9_PARPJ	Paraburkholderia phytofirmans PsJN	3635	2.70E-31	122.4	69.8	4	14	0.0013	2400	11.60	0.17	111	212	1373	1472	1366	1491	0.76	YadA domain protein	0
B2SYM9_PARPJ	Paraburkholderia phytofirmans PsJN	3635	2.70E-31	122.4	69.8	11	14	0.0013	2400	11.64	0.1	112	212	2358	2456	2350	2475	0.73	YadA domain protein	0
A0A3E0CN26_9BURK	Paraburkholderia sp. BL6669N4	3173	1.00E-23	97.7	50.6	3	9	0.0013	2400	11.59	0.25	110	212	1545	1651	1537	1668	0.82	Trimeric autotransporter adhesin	0
A0A3E0CN26_9BURK	Paraburkholderia sp. BL6669N6	3173	1.00E-23	97.7	50.6	5	9	0.0013	2400	11.59	0.25	110	212	1845	1951	1837	1968	0.82	Trimeric autotransporter adhesin	0
A0A3D9MBL0_9BURK	Paraburkholderia sp. BL27I4N3	3204	8.10E-17	75.2	76.3	1	10	0.0013	2400	11.59	0.1	126	214	506	595	484	612	0.79	Trimeric autotransporter adhesin	0
A0A3D9MBL0_9BURK	Paraburkholderia sp. BL27I4N3	3204	8.10E-17	75.2	76.3	3	10	0.0013	2400	11.59	0.1	126	214	1014	1103	992	1120	0.79	Trimeric autotransporter adhesin	0
I2IG27_9BURK	Burkholderia sp. Ch1-1	1826	5.70E-10	52.8	23.2	3	5	0.0013	2400	11.62	0.03	126	212	459	548	420	566	0.82	Hemagglutinin-like protein (Fragment)	0
A0A4Y8N995_9BURK	Paraburkholderia dipogonis	2890	2.00E-07	44.5	47.8	2	4	0.0013	2500	11.57	0.2	144	212	1431	1501	1401	1521	0.76	Adhesin	0
A0A3D9MBL0_9BURK	Paraburkholderia sp. BL27I4N3	3204	8.10E-17	75.2	76.3	10	10	0.0015	2800	11.37	0.07	122	228	2546	2659	2504	2662	0.77	Trimeric autotransporter adhesin	0
A0A3E0CN26_9BURK	Paraburkholderia sp. BL6669N10	3173	1.00E-23	97.7	50.6	9	9	0.0016	2900	11.36	0.05	122	228	2516	2629	2476	2632	0.77	Trimeric autotransporter adhesin	0
B2SYM9_PARPJ	Paraburkholderia phytofirmans PsJN	3635	2.70E-31	122.4	69.8	9	14	0.0016	3000	11.31	0.08	142	212	2102	2175	2070	2197	0.7	YadA domain protein	0
B2SYM9_PARPJ	Paraburkholderia phytofirmans PsJN	3635	2.70E-31	122.4	69.8	12	14	0.0017	3100	11.24	0.17	142	212	2527	2600	2496	2619	0.73	YadA domain protein	0
A0A5Q4Z2J2_9BURK	Paraburkholderia sp. Msb3	3475	9.10E-21	88.1	47.5	1	6	0.0018	3300	11.17	0.15	123	212	377	469	358	487	0.75	Putative membrane-anchored cell surface protein, haemagluttinin	0
Q13U92_PARXL	Paraburkholderia xenovorans (strain LB400)	4726	2.00E-30	119.5	86.6	5	14	0.0021	3900	10.94	0.39	103	212	2010	2119	1975	2137	0.78	Putative membrane-anchored cell surface protein, haemagluttinin	0
Q13U92_PARXL	Paraburkholderia xenovorans (strain LB400)	4726	2.00E-30	119.5	86.6	11	14	0.0021	3900	10.92	0.63	103	212	2916	3026	2878	3044	0.77	Putative membrane-anchored cell surface protein, haemagluttinin	0
A0A4R1UP80_9BURK	Paraburkholderia sp. GV060	3796	5.70E-20	85.5	85.2	8	11	0.0022	4100	10.85	0.38	109	212	2363	2470	2355	2491	0.8	Trimeric autotransporter adhesin	0
I2IG27_9BURK	Burkholderia sp. Ch1-1	1826	5.70E-10	52.8	23.2	4	5	0.0023	4300	10.78	0.3	111	211	593	691	584	709	0.73	Hemagglutinin-like protein (Fragment)	0
A0A3D9MBL0_9BURK	Paraburkholderia sp. BL27I4N3	3204	8.10E-17	75.2	76.3	7	10	0.0026	4700	10.65	0.16	143	212	1754	1826	1726	1843	0.78	Trimeric autotransporter adhesin	0
A0A5Q4Z2J2_9BURK	Paraburkholderia sp. Msb3	3475	9.10E-21	88.1	47.5	5	6	0.0026	4800	10.64	0.08	125	212	2108	2198	2029	2215	0.84	Putative membrane-anchored cell surface protein, haemagluttinin	0
Q13U92_PARXL	Paraburkholderia xenovorans (strain LB400)	4726	2.00E-30	119.5	86.6	2	14	0.0027	4900	10.60	0.08	146	212	843	912	827	930	0.78	Putative membrane-anchored cell surface protein, haemagluttinin	0
Q13U92_PARXL	Paraburkholderia xenovorans (strain LB400)	4726	2.00E-30	119.5	86.6	7	14	0.0029	5300	10.49	0.15	145	212	2333	2403	2303	2424	0.77	Putative membrane-anchored cell surface protein, haemagluttinin	0
Q13U92_PARXL	Paraburkholderia xenovorans (strain LB400)	4726	2.00E-30	119.5	86.6	13	14	0.0029	5300	10.49	0.16	145	212	3242	3312	3212	3333	0.77	Putative membrane-anchored cell surface protein, haemagluttinin	0
Q13U92_PARXL	Paraburkholderia xenovorans (strain LB400)	4726	2.00E-30	119.5	86.6	14	14	0.0029	5300	10.49	0.16	145	212	3660	3730	3630	3751	0.77	Putative membrane-anchored cell surface protein, haemagluttinin	0
A0A4R1UP80_9BURK	Paraburkholderia sp. GV060	3796	5.70E-20	85.5	85.2	9	11	0.0029	5300	10.49	0.3	110	223	2514	2630	2507	2637	0.83	Trimeric autotransporter adhesin	0
A0A4R1UP80_9BURK	Paraburkholderia sp. GV060	3796	5.70E-20	85.5	85.2	10	11	0.0029	5300	10.49	0.3	109	212	2663	2770	2655	2789	0.82	Trimeric autotransporter adhesin	0
A0A4R8K685_9BURK	Paraburkholderia sp. BL6665CI2N2	2692	1.60E-19	84	42	3	7	0.003	5500	10.43	0.09	146	212	1308	1378	1292	1396	0.75	Trimeric autotransporter adhesin	0
A0A3D9MBL0_9BURK	Paraburkholderia sp. BL27I4N3	3204	8.10E-17	75.2	76.3	5	10	0.0031	5600	10.40	0.17	124	212	1303	1394	1284	1411	0.75	Trimeric autotransporter adhesin	0
Q13U92_PARXL	Paraburkholderia xenovorans (strain LB400)	4726	2.00E-30	119.5	86.6	3	14	0.0031	5700	10.39	0.11	144	212	1194	1264	1161	1282	0.79	Putative membrane-anchored cell surface protein, haemagluttinin	0
A0A1H5DYA5_9BURK	Burkholderia sp. WP9	4846	1.30E-15	71.2	66.2	6	9	0.0032	5800	10.35	0.13	147	212	3224	3292	3192	3312	0.79	Head domain of trimeric autotransporter adhesin	0
A0A4R8K685_9BURK	Paraburkholderia sp. BL6665CI2N2	2692	1.60E-19	84	42	7	7	0.0033	6000	10.31	0.07	142	212	2009	2081	1977	2099	0.74	Trimeric autotransporter adhesin	0
A0A4Y8N995_9BURK	Paraburkholderia dipogonis	2890	2.00E-07	44.5	47.8	3	4	0.0033	6000	10.30	0.1	111	212	1676	1774	1668	1792	0.76	Adhesin	0
B2SYM9_PARPJ	Paraburkholderia phytofirmans PsJN	3635	2.70E-31	122.4	69.8	1	14	0.0033	6100	10.29	0.16	111	212	679	778	670	797	0.75	YadA domain protein	0
B2SYM9_PARPJ	Paraburkholderia phytofirmans PsJN	3635	2.70E-31	122.4	69.8	5	14	0.0033	6100	10.28	0.17	111	212	1517	1616	1510	1635	0.75	YadA domain protein	0
A0A1H5DYA5_9BURK	Burkholderia sp. WP9	4846	1.30E-15	71.2	66.2	7	9	0.0034	6200	10.25	0.15	137	210	3493	3568	3484	3590	0.77	Head domain of trimeric autotransporter adhesin	0
A0A1H5DYA5_9BURK	Burkholderia sp. WP9	4846	1.30E-15	71.2	66.2	3	9	0.0034	6300	10.25	0.1	143	217	2609	2685	2579	2699	0.78	Head domain of trimeric autotransporter adhesin	0
B2SYM9_PARPJ	Paraburkholderia phytofirmans PsJN	3635	2.70E-31	122.4	69.8	6	14	0.0036	6600	10.19	0.25	148	212	1683	1751	1668	1773	0.73	YadA domain protein	0
A0A4R1UP80_9BURK	Paraburkholderia sp. GV060	3796	5.70E-20	85.5	85.2	3	11	0.0039	7200	10.05	0.35	110	217	1220	1330	1212	1345	0.8	Trimeric autotransporter adhesin	0

A0A1H5DYA5_9BURK	Burkholderia sp. WP9	4846	1.30E-15	71.2	66.2	4	9	0.004	7200	10.04	0.09	136	212	2927	3006	2918	3027	0.78	Head domain of trimeric autotransporter adhesin	0
Q13U92_PARXL	Paraburkholderia xenovorans (strain LB400)	4726	2.00E-30	119.5	86.6	8	14	0.0042	7600	9.97	0.17	145	212	2476	2546	2448	2565	0.81	Putative membrane-anchored cell surface protein, haemagluttinin	0
I2IG27_9BURK	Burkholderia sp. Ch1-1	1826	5.70E-10	52.8	23.2	1	5	0.0041	7600	9.98	0.14	146	212	31	100	8	122	0.78	Hemagglutinin-like protein (Fragment)	0
B2SYM9_PARPJ	Paraburkholderia phytofirmans PsJN	3635	2.70E-31	122.4	69.8	8	14	0.0043	7900	9.93	0.2	148	212	1963	2031	1950	2051	0.76	YadA domain protein	0
A0A4R1UP80_9BURK	Paraburkholderia sp. GV060	3796	5.70E-20	85.5	85.2	1	11	0.0044	8000	9.90	0.3	110	212	927	1033	919	1052	0.81	Trimeric autotransporter adhesin	0
A0A4R1UP80_9BURK	Paraburkholderia sp. GV060	3796	5.70E-20	85.5	85.2	5	11	0.0047	8700	9.78	0.24	110	212	1663	1769	1656	1788	0.8	Trimeric autotransporter adhesin	0
A0A4R1UP80_9BURK	Paraburkholderia sp. GV060	3796	5.70E-20	85.5	85.2	4	11	0.0049	9000	9.74	0.21	110	223	1513	1629	1506	1637	0.83	Trimeric autotransporter adhesin	0
A0A5Q4Z2J2_9BURK	Paraburkholderia sp. Msb3	3475	9.10E-21	88.1	47.5	4	6	0.0051	9400	9.68	0.17	143	212	1678	1750	1652	1767	0.81	Putative membrane-anchored cell surface protein, haemagluttinin	0
A0A5Q4Z2J2_9BURK	Paraburkholderia sp. Msb3	3475	9.10E-21	88.1	47.5	6	6	0.0051	9400	9.68	0.11	112	211	2244	2341	2234	2360	0.77	Putative membrane-anchored cell surface protein, haemagluttinin	0
A0A4Y8N995_9BURK	Paraburkholderia dipogonis	2890	2.00E-07	44.5	47.8	1	4	0.0054	9900	9.60	0.22	144	214	537	609	507	626	0.75	Adhesin	0
Q13U92_PARXL	Paraburkholderia xenovorans (strain LB400)	4726	2.00E-30	119.5	86.6	4	14	0.0055	10000	9.58	0.1	144	212	1855	1926	1825	1944	0.78	Putative membrane-anchored cell surface protein, haemagluttinin	0
A0A6F9A3I3_9TELE	Coregonus sp. 'balchen	1115	4.80E-12	59.6	1	2	6	0.0056	10000	9.54	0.01	98	189	175	259	138	265	0.65	Uncharacterized protein	0
Q13U92_PARXL	Paraburkholderia xenovorans (strain LB400)	4726	2.00E-30	119.5	86.6	9	14	0.006	11000	9.45	0.11	144	212	2618	2689	2588	2710	0.77	Putative membrane-anchored cell surface protein, haemagluttinin	0
Q13U92_PARXL	Paraburkholderia xenovorans (strain LB400)	4726	2.00E-30	119.5	86.6	10	14	0.0057	11000	9.51	0.16	145	212	2762	2832	2734	2850	0.81	Putative membrane-anchored cell surface protein, haemagluttinin	0
A0A318FGQ1_KLEOX	Klebsiella oxytoca	1845	0.0031	30.8	2.7	1	1	6.20E-03	11000	9.41	0.05	117	227	443	559	437	563	0.81	Autotransporter adhesin (Fragment)	0
I2IG26_9BURK	Burkholderia sp. Ch1-1	2342	2.10E-09	51	16.3	4	5	0.0064	12000	9.35	0.09	142	211	1838	1909	1801	1930	0.73	Hemagglutinin-like protein (Fragment)	0
A0A3E0CN26_9BURK	Paraburkholderia sp. BL6669N2	3173	1.00E-23	97.7	50.6	1	9	0.0073	13000	9.16	0.16	127	212	1264	1351	1241	1368	0.79	Trimeric autotransporter adhesin	0
A0A4Y8N995_9BURK	Paraburkholderia dipogonis	2890	2.00E-07	44.5	47.8	4	4	0.0072	13000	9.19	0.06	113	212	1951	2047	1942	2065	0.73	Adhesin	0
A0A495GKR6_9BURK	Paraburkholderia sp. BL17N1	1417	5.10E-11	56.2	15.9	4	5	0.0075	14000	9.14	0.13	142	214	1032	1106	1004	1123	0.76	Trimeric autotransporter adhesin	0
Q13U92_PARXL	Paraburkholderia xenovorans (strain LB400)	4726	2.00E-30	119.5	86.6	1	14	0.0081	15000	9.02	0.06	146	211	362	430	345	451	0.79	Putative membrane-anchored cell surface protein, haemagluttinin	0
A0A495GKR6_9BURK	Paraburkholderia sp. BL17N1	1417	5.10E-11	56.2	15.9	1	5	0.0081	15000	9.03	0.03	124	208	288	375	267	399	0.76	Trimeric autotransporter adhesin	0
A0A2T0Q1E9_9BURK	Paraburkholderia sp. BL25I1N1	1498	4.50E-08	46.6	24.8	4	4	0.008	15000	9.04	0.13	142	214	1260	1334	1232	1351	0.76	Trimeric autotransporter adhesin (Fragment)	0
A0A1H5DYA5_9BURK	Burkholderia sp. WP9	4846	1.30E-15	71.2	66.2	8	9	0.0089	16000	8.89	0.15	142	211	3641	3713	3614	3737	0.76	Head domain of trimeric autotransporter adhesin	0
B2SYM9_PARPJ	Paraburkholderia phytofirmans PsJN	3635	2.70E-31	122.4	69.8	2	14	0.0091	17000	8.87	0.17	142	212	1113	1186	1080	1205	0.73	YadA domain protein	0
A0A6F9A3I3_9TELE	Coregonus sp. 'balchen	1115	4.80E-12	59.6	1	5	6	0.0094	17000	8.82	0.02	115	189	722	790	679	796	0.75	Uncharacterized protein	0
B2SYM9_PARPJ	Paraburkholderia phytofirmans PsJN	3635	2.70E-31	122.4	69.8	10	14	0.0098	18000	8.75	0.15	148	212	2243	2312	2230	2332	0.75	YadA domain protein	0
A0A3E0CN26_9BURK	Paraburkholderia sp. BL6669N8	3173	1.00E-23	97.7	50.6	7	9	0.0099	18000	8.75	0.17	144	212	2174	2245	2144	2262	0.78	Trimeric autotransporter adhesin	0
A0A5Q4Z2J2_9BURK	Paraburkholderia sp. Msb3	3475	9.10E-21	88.1	47.5	2	6	0.01	18000	8.72	0.09	142	211	790	861	753	882	0.73	Putative membrane-anchored cell surface protein, haemagluttinin	0
A0A1H5DYA5_9BURK	Burkholderia sp. WP9	4846	1.30E-15	71.2	66.2	5	9	0.0097	18000	8.78	0.11	146	212	3080	3149	3048	3167	0.79	Head domain of trimeric autotransporter adhesin	0
A0A6F9A3I3_9TELE	Coregonus sp. 'balchen	1115	4.80E-12	59.6	1	6	6	0.01	19000	8.66	0.01	116	189	908	975	855	981	0.67	Uncharacterized protein	0
I2IG26_9BURK	Burkholderia sp. Ch1-1	2342	2.10E-09	51	16.3	1	5	0.011	19000	8.65	0.05	142	212	648	720	611	740	0.72	Hemagglutinin-like protein (Fragment)	0
A0A6F9A3I3_9TELE	Coregonus sp. 'balchen	1115	4.80E-12	59.6	1	4	6	0.011	20000	8.57	0.02	115	189	545	613	506	619	0.77	Uncharacterized protein	0
A0A6F9A3I3_9TELE	Coregonus sp. 'balchen	1115	4.80E-12	59.6	1	3	6	0.012	21000	8.52	0.02	116	189	369	436	331	442	0.78	Uncharacterized protein	0
A0A4R8K685_9BURK	Paraburkholderia sp. BL6665CI2N2	2692	1.60E-19	84	42	2	7	0.015	28000	8.14	0.09	124	215	1151	1244	1131	1264	0.7	Trimeric autotransporter adhesin	0
B2SYM9_PARPJ	Paraburkholderia phytofirmans PsJN	3635	2.70E-31	122.4	69.8	14	14	0.016	29000	8.09	0.16	112	212	2926	3024	2918	3044	0.72	YadA domain protein	0
I2IG27_9BURK	Burkholderia sp. Ch1-1	1826	5.70E-10	52.8	23.2	2	5	0.016	30000	8.05	0.09	127	216	222	313	161	328	0.83	Hemagglutinin-like protein (Fragment)	0
A0A1H5DYA5_9BURK	Burkholderia sp. WP9	4846	1.30E-15	71.2	66.2	2	9	0.017	31000	8.00	0.12	142	226	2307	2392	2276	2396	0.77	Head domain of trimeric autotransporter adhesin	0

A0A1H5DYA5_9BURK	Burkholderia sp. WP9	4846	1.30E-15	71.2	66.2	1	9	0.018	32000	7.92	0.2	137	225	984	1073	975	1080	0.75	Head domain of trimeric autotransporter adhesin	0
A0A4R1UP80_9BURK	Paraburkholderia sp. GV060	3796	5.70E-20	85.5	85.2	2	11	0.018	33000	7.91	0.21	142	212	1104	1176	1069	1198	0.69	Trimeric autotransporter adhesin	0
A0A4R1UP80_9BURK	Paraburkholderia sp. GV060	3796	5.70E-20	85.5	85.2	6	11	0.019	34000	7.84	0.19	142	212	1840	1912	1805	1931	0.72	Trimeric autotransporter adhesin	0
I2IG27_9BURK	Burkholderia sp. Ch1-1	1826	5.70E-10	52.8	23.2	5	5	0.019	34000	7.84	0.16	143	215	1176	1250	1150	1265	0.75	Hemagglutinin-like protein (Fragment)	0
A0A3D9MBL0_9BURK	Paraburkholderia sp. BL27I4N3	3204	8.10E-17	75.2	76.3	2	10	0.019	35000	7.80	0.13	142	214	664	738	636	755	0.76	Trimeric autotransporter adhesin	0
A0A3D9MBL0_9BURK	Paraburkholderia sp. BL27I4N3	3204	8.10E-17	75.2	76.3	4	10	0.019	35000	7.80	0.13	142	214	1172	1246	1144	1263	0.76	Trimeric autotransporter adhesin	0
A0A4R1UP80_9BURK	Paraburkholderia sp. GV060	3796	5.70E-20	85.5	85.2	7	11	0.024	44000	7.49	0.2	110	213	2085	2185	2078	2206	0.73	Trimeric autotransporter adhesin	0
A0A4R8K685_9BURK	Paraburkholderia sp. BL6665CI2N2	2692	1.60E-19	84	42	1	7	0.025	45000	7.45	0.14	111	214	561	660	553	679	0.71	Trimeric autotransporter adhesin	0
A0A3D9MBL0_9BURK	Paraburkholderia sp. BL27I4N3	3204	8.10E-17	75.2	76.3	8	10	0.025	45000	7.45	0.13	124	212	2030	2122	2010	2139	0.74	Trimeric autotransporter adhesin	0
A0A661J770_9DELT	Deltaproteobacteria bacterium	643	0.0002	34.7	9.7	1	1	2.60E-02	48000	7.37	0.08	85	134	512	560	492	602	0.79	Vault domain-containing protein (Fragment)	0
Q13U92_PARXL	Paraburkholderia xenovorans (strain LB400)	4726	2.00E-30	119.5	86.6	6	14	0.026	49000	7.35	0.14	145	212	2190	2260	2160	2277	0.79	Putative membrane-anchored cell surface protein, haemagluttinin	0
A0A6F9A3I3_9TELE	Coregonus sp. 'balchen	1115	4.80E-12	59.6	1	1	6	0.027	49000	7.33	0.01	150	189	35	74	10	80	0.87	Uncharacterized protein	0
I2IG26_9BURK	Burkholderia sp. Ch1-1	2342	2.10E-09	51	16.3	2	5	0.027	49000	7.33	0.12	144	220	920	997	890	1010	0.69	Hemagglutinin-like protein (Fragment)	0
A0A4R8K685_9BURK	Paraburkholderia sp. BL6665CI2N2	2692	1.60E-19	84	42	4	7	0.028	51000	7.27	0.06	148	212	1446	1515	1433	1533	0.75	Trimeric autotransporter adhesin	0
Q13U92_PARXL	Paraburkholderia xenovorans (strain LB400)	4726	2.00E-30	119.5	86.6	12	14	0.028	52000	7.25	0.16	145	212	3099	3169	3071	3186	0.8	Putative membrane-anchored cell surface protein, haemagluttinin	0

*Query: miEBLP-4 in *Miniopterus fuliginosus* (LC708263.1:1479-2180)

Table S3. TFASTX search hits using miEBLX-4 in *Miniopterus schreibersii* as query.

Subject accession	Subject species	Subject order	Subject class	% Identity	Alignment length	Query start*	Query end*	Subject start	Subject end	E value	Bit score	Definition
NW_015504245.1	*Miniopterus natalensis*	Chiroptera	Mammalia	95.41	109	1	109	192924	193250	8.40E-52	207.3	miEBLX/P-4
PVJG01006939.1	*Miniopterus schreibersii*	Chiroptera	Mammalia	100.00	109	1	109	45354	45028	3.90E-47	194.7	miEBLX/P-4
NW_015504783.1	*Miniopterus natalensis*	Chiroptera	Mammalia	73.79	108	4	109	198018	197716	6.30E-31	137.9	miEBLN/X/P-2
NW_015504642.1	*Miniopterus natalensis*	Chiroptera	Mammalia	71.03	108	2	109	757737	758057	1.90E-30	134.7	miEBLN/X/P-3
PVJG01014896.1	*Miniopterus schreibersii*	Chiroptera	Mammalia	70.09	108	2	109	29054	28734	5.80E-25	119.7	miEBLN/X/P-3
PVJG01000137.1	*Miniopterus schreibersii*	Chiroptera	Mammalia	71.84	108	4	109	104055	104357	1.60E-24	120.4	miEBLN/X/P-2
NW_015504659.1	*Miniopterus natalensis*	Chiroptera	Mammalia	59.41	113	1	109	442538	442248	9.00E-13	77.6	miEBLN/X/P-1
PVJG01001939.1	*Miniopterus schreibersii*	Chiroptera	Mammalia	60.61	111	1	109	113354	113064	4.70E-12	79	miEBLN/X/P-1
NW_025333895.1	*Phyllostomus hastatus*	Chiroptera	Mammalia	42.72	107	4	108	435208	435515	9.10E-12	74.4	mpEBLX/P
NC_040905.2	*Phyllostomus discolor*	Chiroptera	Mammalia	41.75	107	4	108	205140494	205140801	6.40E-10	68.3	mpEBLX/P
PVKU01001816.1	*Anoura caudifer*	Chiroptera	Mammalia	35.24	105	4	108	132409	132723	9.20E-09	69.1	mpEBLX/P
PVKM010001206.1	*Carollia perspicillata*	Chiroptera	Mammalia	41.18	103	4	104	23878	24182	2.20E-08	69.4	mpEBLX/P
PVIA01006371.1	*Tonatia saurophila*	Chiroptera	Mammalia	41.90	107	4	108	95618	95926	4.00E-08	65.9	mpEBLX/P
NW_023533648.1	*Artibeus jamaicensis*	Chiroptera	Mammalia	39.42	105	5	108	6183493	6183184	6.30E-08	64	mpEBLX/P
PVJD01000578.1	*Mormoops blainvillei*	Chiroptera	Mammalia	42.31	107	4	108	282597	282280	3.80E-07	63	mpEBLX/P
JAIWKQ010000315.1	*Pteronotus parnellii mesoamericanus*	Chiroptera	Mammalia	40.59	104	8	108	9949490	9949186	3.10E-06	56	mpEBLX/P
JAIWKQ010000019.1	*Pteronotus parnellii mesoamericanus*	Chiroptera	Mammalia	35.85	107	4	108	1349954	1350270	1.30E-05	54	-
NC_045765.1	*Phocoena sinus*	Artiodactyla	Mammalia	37.50	73	24	96	1188004	1188219	1.30E-05	54.2	-
LR991643.1	*Trachurus trachurus*	Carangiformes	Actinopterygii	33.33	101	10	107	23969500	23969209	3.90E-05	51	-
NC_031975.2	*Oreochromis niloticus*	Cichliformes	Actinopterygii	35.35	111	5	109	32600298	32600612	4.00E-05	51.7	-
PVJI01003473.1	*Micronycteris hirsuta*	Chiroptera	Mammalia	39.80	105	4	108	95456	95749	5.70E-05	56.9	mpEBLX/P
NC_051213.1	*Pygocentrus nattereri*	Characiformes	Actinopterygii	29.35	92	17	108	21484659	21484934	9.20E-05	50.4	-

*Query: miEBX-4 in *Miniopterus schreibersii (* PVJG01006939.1:45354-45028)

Table S4. TFASTY search hits using miEBLX-4 in *Miniopterus schreibersii* as query.

Subject accession	Subject species	Subject order	Subject class	% Identity	Alignment length	Query start*	Query end*	Subject start	Subject end	E value	Bit score	Definition
NW_015504245.1	*Miniopterus natalensis*	Chiroptera	Mammalia	95.41	109	1	109	192924	193247	3.80E-43	178.5	miEBLX/P-4
PVJG01006939.1	*Miniopterus schreibersii*	Chiroptera	Mammalia	100.00	109	1	109	45354	45031	4.60E-42	177.9	miEBLX/P-4
NW_015504783.1	*Miniopterus natalensis*	Chiroptera	Mammalia	75.73	108	4	109	198018	197719	8.80E-27	124.2	miEBLN/X/P-2
NW_015504642.1	*Miniopterus natalensis*	Chiroptera	Mammalia	71.03	108	2	109	757737	758054	2.80E-25	117.5	miEBLN/X/P-3
PVJG01000137.1	*Miniopterus schreibersii*	Chiroptera	Mammalia	73.79	108	4	109	104055	104354	9.20E-23	114.6	miEBLN/X/P-2
PVJG01014896.1	*Miniopterus schreibersii*	Chiroptera	Mammalia	70.09	108	2	109	29054	28737	3.50E-22	110.5	miEBLN/X/P-3
NW_015504659.1	*Miniopterus natalensis*	Chiroptera	Mammalia	62.38	113	1	109	442538	442251	2.90E-11	72.6	miEBLN/X/P-1
PVJG01001939.1	*Miniopterus schreibersii*	Chiroptera	Mammalia	62.63	111	1	109	113354	113067	6.50E-11	75.3	miEBLN/X/P-1
NW_025333895.1	*Phyllostomus hastatus*	Chiroptera	Mammalia	43.27	107	4	108	435208	435512	1.20E-09	67.4	mpEBLX/P
NC_040905.2	*Phyllostomus discolor*	Chiroptera	Mammalia	42.31	107	4	108	205140494	205140798	4.10E-08	62.3	mpEBLX/P
PVKU01001816.1	*Anoura caudifer*	Chiroptera	Mammalia	35.24	105	4	108	132409	132720	6.80E-08	66.2	mpEBLX/P
PVKM010001206.1	*Carollia perspicillata*	Chiroptera	Mammalia	41.18	103	4	104	23878	24179	1.10E-07	67.1	mpEBLX/P
PVIA01006371.1	*Tonatia saurophila*	Chiroptera	Mammalia	42.86	107	4	108	95618	95923	4.80E-07	62.3	mpEBLX/P
NW_023533648.1	*Artibeus jamaicensis*	Chiroptera	Mammalia	39.42	105	5	108	6183493	6183187	8.40E-07	60.2	mpEBLX/P
PVJD01000578.1	*Mormoops blainvillei*	Chiroptera	Mammalia	42.06	108	4	108	282597	282283	2.80E-06	60.1	mpEBLX/P
JAIWKQ010000315.1	*Pteronotus parnellii mesoamericanus*	Chiroptera	Mammalia	41.58	104	8	108	9949490	9949189	8.40E-06	54.6	mpEBLX/P
JAIWKQ010000019.1	*Pteronotus parnellii mesoamericanus*	Chiroptera	Mammalia	36.79	107	4	108	1349954	1350267	3.40E-05	52.6	-
CM026086.1	*Anableps anableps*	Cyprinodontiformes	Actinopterygii	29.89	93	7	92	26514181	26513908	4.60E-05	50.9	-

*Query: miEBLX-4 in *Miniopterus schreibersii* (PVJG01006939.1:45354-45028)

Table S5. TFASTX search hits using miEBLP-4 in *Miniopterus schreibersii* as query.

Subject accession	Subject species	Subject order	Subject class	% Identity	Alignment length	Query start*	Query end*	Subject start	Subject end	E value	Bit score	Definition
NW_015504245.1	*Miniopterus natalensis*	Chiroptera	Mammalia	96.57	234	1	233	192979	193675	5.00E-103	378.6	miEBLX/P-4
PVJG01006939.1	*Miniopterus schreibersii*	Chiroptera	Mammalia	100.00	233	1	233	45299	44601	1.20E-94	353.7	miEBLX/P-4
NW_015504642.1	*Miniopterus natalensis*	Chiroptera	Mammalia	72.17	235	3	233	757795	758468	7.90E-65	250	miEBLN/X/P-3
PVJG01014896.1	*Miniopterus schreibersii*	Chiroptera	Mammalia	72.61	235	3	233	28996	28323	5.60E-56	223.9	miEBLN/X/P-3
NW_015504783.1	*Miniopterus natalensis*	Chiroptera	Mammalia	65.79	235	2	232	197970	197297	1.10E-54	218	miEBLN/X/P-2
NW_015504659.1	*Miniopterus natalensis*	Chiroptera	Mammalia	64.79	221	18	233	442469	441843	4.10E-47	192.8	miEBLN/X/P-1
PVJG01000137.1	*Miniopterus schreibersii*	Rodentia	Mammalia	64.91	235	2	232	104103	104776	7.60E-45	189.1	miEBLN/X/P-2
PVJG01001939.1	*Miniopterus schreibersii*	Chiroptera	Mammalia	64.95	219	18	233	113285	112650	5.60E-43	182.9	miEBLN/X/P-1
NW_017870072.1	*Castor canadensis*	Rodentia	Mammalia	54.42	225	14	232	1653454	1654097	1.20E-38	166	-
NW_020091914.1	*Desmodus rotundus*	Chiroptera	Mammalia	49.75	209	19	226	8982376	8982980	1.60E-34	152.8	mpEBLX/P
NC_040905.2	*Phyllostomus discolor*	Chiroptera	Mammalia	46.40	229	1	226	205140540	205141195	5.90E-32	142.6	mpEBLX/P
NW_025333895.1	*Phyllostomus hastatus*	Chiroptera	Mammalia	47.11	232	1	228	435254	435914	6.70E-31	139.1	mpEBLX/P
JAIWKQ010000315.1	*Pteronotus parnellii mesoamericanus*	Chiroptera	Mammalia	45.70	229	1	226	9949456	9948797	3.90E-30	136.5	mpEBLX/P
NW_023533648.1	*Artibeus jamaicensis*	Chiroptera	Mammalia	42.99	227	1	225	6183450	6182792	1.70E-27	130.1	mpEBLX/P
PVKE010012405.1	*Craseonycteris thonglongyai*	Chiroptera	Mammalia	41.67	236	3	232	5592	6264	5.50E-27	130.5	ctEBL/X/P
JAIWKQ010000019.1	*Pteronotus parnellii mesoamericanus*	Chiroptera	Mammalia	40.36	244	1	233	1350000	1350687	1.70E-25	121.2	-
PVKM010001206.1	*Carollia perspicillata*	Chiroptera	Mammalia	44.44	233	1	226	23924	24595	6.60E-25	125.4	mpEBLX/P
PVJD01000578.1	*Mormoops blainvillei*	Chiroptera	Mammalia	43.48	213	1	209	282551	281938	1.00E-24	122.5	mpEBLX/P
PVIA01006371.1	*Tonatia saurophila*	Chiroptera	Mammalia	44.09	228	1	226	95664	96317	2.00E-24	121.2	mpEBLX/P
VMDR010008610.1	*Macrotus californicus*	Chiroptera	Mammalia	45.00	227	5	226	13930	13280	2.30E-24	121.6	mpEBLX/P
PVJI01003473.1	*Micronycteris hirsuta*	Chiroptera	Mammalia	44.95	231	4	232	95511	96158	2.30E-23	119.1	mpEBLX/P
VZQC01011341.1	*Rhizomys pruinosus*	Rodentia	Mammalia	39.04	237	1	233	2839825	2839142	3.10E-23	115.1	-
PVKU01001816.1	*Anoura caudifer*	Chiroptera	Mammalia	42.29	235	3	232	132461	133137	2.40E-21	112	mpEBLX/P
PVJD01014513.1	*Mormoops blainvillei*	Chiroptera	Mammalia	37.00	238	3	233	24523	23848	3.50E-20	105	-
NW_023530264.1	*Sturnira hondurensis*	Chiroptera	Mammalia	40.10	228	1	226	282817	283429	2.90E-17	95.3	mpEBLX/P
NW_008360317.1	*Nannospalax galili*	Rodentia	Mammalia	38.10	243	3	233	1026867	1027541	3.60E-17	97.1	-
PVKE010000102.1	*Craseonycteris thonglongyai*	Chiroptera	Mammalia	47.85	173	64	230	35997	35519	5.50E-17	99.3	-
NW_024870852.1	*Dipodomys spectabilis*	Rodentia	Mammalia	47.77	162	8	164	7861406	7860933	1.90E-17	94.9	-
JAJVDE010000074.1	*Calyptommatus sinebrachiatus*	Squamata	Reptilia	37.29	194	30	214	925172	925722	7.20E-16	89.8	-
NC_063166.1	*Perognathus longimembris pacificus*	Rodentia	Mammalia	39.20	207	27	229	64882957	64882361	1.30E-15	88.8	-
PVHN010009826.1	*Dipodomys stephensi*	Rodentia	Mammalia	50.34	156	8	157	34184	34625	2.80E-13	85.6	-
JADPQB010004081.1	*Crotalus adamanteus*	Squamata	Reptilia	32.00	190	28	212	322512	323051	2.10E-12	78	-
NW_024096970.1	*Crotalus tigris*	Squamata	Reptilia	32.37	192	28	212	3698000	3698539	2.10E-12	77.6	-
NW_015505136.1	*Miniopterus natalensis*	Chiroptera	Mammalia	44.12	103	131	232	4261937	4262245	2.70E-11	73.8	-
CM034703.1	*Phrynosoma platyrhinos*	Squamata	Reptilia	28.08	223	21	233	147596247	147596880	4.40E-11	73.6	-
JPMF01384358.1	*Crotalus pyrrhus*	Squamata	Reptilia	31.82	191	28	212	623	85	1.20E-10	69.1	-
CM012306.1	*Crotalus viridis viridis*	Squamata	Reptilia	31.82	191	28	212	221015586	221016126	1.30E-10	71.8	-
LR699156.1	*Geotrypetes seraphini*	Gymnophiona	Amphibia	36.09	182	57	233	3490908	3490387	1.50E-09	69	-
NC_047094.1	*Geotrypetes seraphini*	Gymnophiona	Amphibia	36.09	182	57	233	3490908	3490387	1.50E-09	69	-
JAJVDE010000029.1	*Calyptommatus sinebrachiatus*	Squamata	Reptilia	33.71	190	40	213	1645108	1645666	2.10E-09	68.4	-
LVCR01018846.1	*Crotalus horridus*	Squamata	Reptilia	32.39	188	28	212	1815	2346	2.80E-09	69.3	-
NW_008350555.1	*Nannospalax galili*	Rodentia	Mammalia	36.61	208	1	206	1910872	1910318	4.70E-09	70.1	-
RJWH01000001.1	*Solenodon paradoxus*	Eulipotyphla	Mammalia	40.43	94	140	232	2816798	2817075	4.10E-08	67.1	-
NW_020066339.1	*Desmodus rotundus*	Chiroptera	Mammalia	50.00	65	162	226	14	205	5.30E-08	55.1	-
NW_012267229.1	*Dipodomys ordii*	Rodentia	Mammalia	47.45	142	8	145	11151048	11150631	6.70E-08	65.2	-
PVJG01004446.1	*Miniopterus schreibersii*	Chiroptera	Mammalia	61.29	62	171	232	74465	74280	2.20E-07	64.3	-
JAJFZI010455917.1	*Anilios bituberculatus*	Squamata	Reptilia	30.88	238	7	228	129	788	5.30E-07	58.8	-
PVKD010003509.1	*Cricetomys gambianus*	Rodentia	Mammalia	37.42	178	11	185	99215	99693	2.00E-05	60.8	-
JAHWGE010699523.1	*Chrysopelea ornata*	Squamata	Reptilia	34.38	177	29	201	99	583	6.60E-05	51.3	-
LR812501.1	*Danio aesculapii*	Cypriniformes	Actinopterygii	18.37	201	18	215	1352310	1352899	7.10E-05	52.1	-
NDGN011162872.1	*Tympanoctomys barrerae*	Rodentia	Mammalia	24.14	182	30	206	2386	2922	0.00002	55.8	ocEBLX/P

*Query: miEBLP-4 in *Miniopterus schreibersii* (PVJG01006939.1:45299-44601)

Table S6. TFASTY search hits using miEBLP-4 in *Miniopterus schreibersii* as query.

Subject accession	Subject species	Subject order	Subject class	% Identity	Alignment length	Query start*	Query end*	Subject start	Subject end	E value	Bit score	Definition
NW_015504245.1	*Miniopterus natalensis*	Chiroptera	Mammalia	96.57	234	1	233	192979	193672	8.90E-110	401	miEBLX/P-4
PVJG01006939.1	*Miniopterus schreibersii*	Chiroptera	Mammalia	100.00	233	1	233	45299	44604	1.00E-92	347.2	miEBLX/P-4
NW_015504642.1	*Miniopterus natalensis*	Chiroptera	Mammalia	73.04	235	3	233	757795	758465	2.70E-70	268.1	miEBLN/X/P-3
NW_015504783.1	*Miniopterus natalensis*	Chiroptera	Mammalia	66.23	235	2	232	197970	197300	6.70E-59	231.9	miEBLN/X/P-2
PVJG01014896.1	*Miniopterus schreibersii*	Chiroptera	Mammalia	73.48	235	3	233	28996	28326	1.10E-55	223	miEBLN/X/P-3
NW_015504659.1	*Miniopterus natalensis*	Chiroptera	Mammalia	65.73	221	18	233	442469	441846	5.60E-52	209	miEBLN/X/P-1
NW_017870072.1	*Castor canadensis*	Rodentia	Mammalia	55.35	225	14	232	1653454	1654094	4.00E-48	197.5	-
PVJG01000137.1	*Miniopterus schreibersii*	Chiroptera	Mammalia	65.35	235	2	232	104103	104773	2.80E-44	187.2	miEBLN/X/P-2
PVJG01001939.1	*Miniopterus schreibersii*	Chiroptera	Mammalia	65.42	219	18	233	113285	112653	9.90E-43	182.1	miEBLN/X/P-1
NC_040905.2	*Phyllostomus discolor*	Chiroptera	Mammalia	47.30	229	1	226	205140540	205141192	2.70E-37	160.3	mpEBLX/P
NW_020091914.1	*Desmodus rotundus*	Chiroptera	Mammalia	50.74	210	19	226	8982376	8982977	1.20E-35	156.5	mpEBLX/P
NW_025333895.1	*Phyllostomus hastatus*	Chiroptera	Mammalia	47.56	232	1	228	435254	435911	5.20E-35	152.8	mpEBLX/P
JAIWKQ010000315.1	*Pteronotus parnellii mesoamericanus*	Chiroptera	Mammalia	46.15	229	1	226	9949456	9948800	7.20E-34	148.9	mpEBLX/P
JAIWKQ010000019.1	*Pteronotus parnellii mesoamericanus*	Chiroptera	Mammalia	38.20	238	1	233	1350000	1350684	3.80E-29	133.2	-
PVKE010012405.1	*Craseonycteris thonglongyai*	Chiroptera	Mammalia	44.16	239	3	232	5592	6261	1.40E-27	132.5	ctEBLX/P
NW_023533648.1	*Artibeus jamaicensis*	Chiroptera	Mammalia	42.99	227	1	225	6183450	6182795	5.00E-27	128.5	mpEBLX/P
VZQC01011341.1	*Rhizomys pruinosus*	Rodentia	Mammalia	39.47	237	1	233	2839825	2839145	7.00E-26	123.9	-
PVIA01006371.1	*Tonatia saurophila*	Chiroptera	Mammalia	44.55	228	1	226	95664	96314	5.20E-25	123.1	mpEBLX/P
PVJD01000578.1	*Mormoops blainvillei*	Chiroptera	Mammalia	44.44	213	1	209	282551	281941	6.10E-25	123.2	mpEBLX/P
PVKM010001206.1	*Carollia perspicillata*	Chiroptera	Mammalia	45.33	233	1	226	23924	24592	7.80E-25	125.2	mpEBLX/P
VMDR010008610.1	*Macrotus californicus*	Chiroptera	Mammalia	45.50	228	5	226	13930	13283	1.10E-24	122.7	mpEBLX/P
PVJI01003473.1	*Micronycteris hirsuta*	Chiroptera	Mammalia	44.95	231	4	232	95511	96155	8.90E-23	117.1	mpEBLX/P
PVKU01001816.1	*Anoura caudifer*	Chiroptera	Mammalia	45.02	238	3	232	132461	133134	6.10E-22	113.9	mpEBLX/P
PVJD01014513.1	*Mormoops blainvillei*	Chiroptera	Mammalia	38.10	237	3	233	24523	23851	1.80E-21	109.3	-
NW_024870852.1	*Dipodomys spectabilis*	Rodentia	Mammalia	47.77	162	8	164	7861406	7.86E+06	3.40E-20	104	-
JAJVDE010000074.1	*Calyptommatus sinebrachiatus*	Squamata	Reptilia	37.85	194	30	214	925172	925719	1.50E-18	98.7	-
NW_008360317.1	*Nannospalax galili*	Rodentia	Mammalia	39.06	244	3	233	1026867	1027538	1.80E-18	101.4	-
NC_063166.1	*Perognathus longimembris pacificus*	Rodentia	Mammalia	39.80	208	27	229	64882957	64882364	8.30E-18	96	-
NW_023530264.1	*Sturnira hondurensis*	Chiroptera	Mammalia	41.06	228	1	226	282817	283426	1.10E-17	96.7	mpEBLX/P
PVKE010000102.1	*Craseonycteris thonglongyai*	Chiroptera	Mammalia	49.08	173	64	230	35997	35522	1.60E-17	101.1	-
NW_024096970.1	*Crotalus tigris*	Squamata	Reptilia	32.37	192	28	212	3698000	3698536	4.70E-16	89.7	-
JADPQB010004081.1	*Crotalus adamanteus*	Squamata	Reptilia	32.00	190	28	212	322512	323048	1.30E-14	85.4	-
PVHN010009826.1	*Dipodomys stephensi*	Rodentia	Mammalia	51.70	156	8	157	34184	34622	1.30E-13	86.7	-
CM034703.1	*Phrynosoma platyrhinos*	Squamata	Reptilia	28.43	214	21	223	147596247	147596862	1.90E-13	81.4	-
NW_015505136.1	*Miniopterus natalensis*	Chiroptera	Mammalia	44.12	103	131	232	4261937	4262242	3.20E-12	76.9	-
JAJVDE010000029.1	*Calyptommatus sinebrachiatus*	Squamata	Reptilia	34.29	190	40	213	1645108	1645663	6.70E-12	76.7	-
CM012306.1	*Crotalus viridis viridis*	Squamata	Reptilia	31.82	191	28	212	221015586	221016123	1.20E-11	75.3	-
JPMF01384358.1	*Crotalus pyrrhus*	Squamata	Reptilia	31.82	191	28	212	623	88	2.70E-10	67.9	-
LR699156.1	*Geotrypetes seraphini*	Gymnophiona	Amphibia	36.09	182	57	233	3490908	3490390	9.40E-10	69.7	-
NC_047094.1	*Geotrypetes seraphini*	Gymnophiona	Amphibia	36.09	182	57	233	3490908	3490390	9.40E-10	69.7	-
NW_012267229.1	*Dipodomys ordii*	Rodentia	Mammalia	48.65	155	8	157	11151048	11150605	1.10E-08	67.8	-
NW_008350555.1	*Nannospalax galili*	Rodentia	Mammalia	36.61	208	1	206	1910872	1910321	1.10E-08	69	-
RJWH01000001.1	*Solenodon paradoxus*	Eulipotyphla	Mammalia	31.76	174	65	232	2816569	2817072	1.30E-08	68.8	-
LVCR01018846.1	*Crotalus horridus*	Squamata	Reptilia	32.39	188	28	212	1815	2343	1.90E-08	66.5	-
NW_020066339.1	*Desmodus rotundus*	Chiroptera	Mammalia	50	65	162	226	14	202	2.70E-08	56.1	-
NW_006278710.1	*Neolamprologus brichardi*	Cichliformes	Actinopterygii	23.08	149	35	182	1021	593	2.90E-07	53.7	-
PVJG01004446.1	*Miniopterus schreibersii*	Chiroptera	Mammalia	61.29	62	171	232	74465	74283	4.00E-07	63.4	-
JAJFZI010455917.1	*Anilios bituberculatus*	Squamata	Reptilia	32.99	236	11	228	144	785	6.70E-07	58.5	-
PVKD010003509.1	*Cricetomys gambianus*	Rodentia	Mammalia	39.26	178	11	185	99215	99690	3.00E-06	63.5	-
NC_059108.1	*Jaculus jaculus*	Rodentia	Mammalia	35.14	157	9	159	16157502	16157072	1.50E-05	55.3	-
NC_051225.1	*Pygocentrus nattereri*	Characiformes	Actinopterygii	22.67	178	3	177	15570500	15569986	2.10E-05	53.6	-
ML237713.1	*Peromyscus polionotus subgriseus*	Rodentia	Mammalia	23.43	194	5	185	3973	3413	2.80E-05	50.2	-
JAHWGE010699523.1	*Chrysopelea ornata*	Squamata	Reptilia	34.38	177	29	201	99	580	3.00E-05	52.4	-
JADMNL010000007.1	*Neoceratodus forsteri*	Dipnoi	Sarcopterygii	30.43	94	5	97	217927847	217927572	3.50E-05	57.7	-
PVKD010006647.1	*Cricetomys gambianus*	Rodentia	Mammalia	35.6	209	11	214	22949	22398	3.60E-05	59.2	-
PVHP010003268.1	*Zapus hudsonius*	Rodentia	Mammalia	40	161	1	155	9598	10043	7.10E-05	58.1	-
NDGN011162872.1	*Tympanoctomys barrerae*	Rodentia	Mammalia	22.99	182	30	206	2386	2919	4.50E-05	54.6	ocEBLX/P

NW_004524665.1	*Octodon degus*	Rodentia	Mammalia	24.31	185	28	206	1886521	1887066	1.30E-05	56	ocEBLX/P

*Query: miEBLP-4 in *Miniopterus schreibersii* (PVJG01006939.1:45299-44601)

Table S7. Primer sequences used in this study.

Primer name	Primer sequence (5' - 3')	Purpose
miEBLN/X/P-1_F	CAGGAGTGACAATGCTGGAAAGAGAG	PCR amplification of miEBLN/X/P-1 locus region in *M. fuliginosus*
miEBLN/X/P-1_R	CTTGCCCAAGGACACACCCAAATAAG	
miEBLN/X/P-2_F	GTACCTAGCCCATCTAACAATGGTATACAC	PCR amplification of miEBLN/X/P-2 locus region in *M. fuliginosus*
miEBLN/X/P-2_R	CAAAGTGTGAGAGTTTCAGGTGCTCCAC	
miEBLN/X/P-3_F	GACAATGGACAGGCTCAGTTAAGGTAAAG	PCR amplification of miEBLN/X/P-3 locus region in *M. fuliginosus*
miEBLN/X/P-3_R	GTTATACACATTCCTATATGGCTCACCAG	
miEBLX/P-4_F	CTCATTTGGGGGGCAGCAGTTTATATTG	PCR amplification of miEBLX/P-4 locus region in *M. fuliginosus*
miEBLX/P-4_R	CAAGACACGTCAGAATAATGAATGGCCTAG	
miEBLN/X/P-1_F0	GTCCCCAACATTTCTTCTTG	Sequencing of PCR-amplified miEBLN/X/P-1 locus region in *M. fuliginosus*
miEBLN/X/P-1_F1	GTCAACCACTGATCTGATTTC	
miEBLN/X/P-1_F2	GTAGAAGTCATCACTGAAGC	
miEBLN/X/P-1_F3	CTCTCAGCCTCTCTCCT	
miEBLN/X/P-1_F4	TGGTGTATATGATGGTGCTTC	
miEBLN/X/P-1_F5	GTGCCAAGCACTGGCAAAA	
miEBLN/X/P-1_F6	GTGTGATACTTATTTGGGGTG	
miEBLN/X/P-1_F7	CAGGATATAGAAGAGCCTGAG	
miEBLN/X/P-1_F8	CTAACACGTGAGTTTGAATCAC	
miEBLN/X/P-1_F9	GTCCAAGTTCCAAGGTGC	
miEBLN/X/P-1_F10	CTCTAGCTATACCTTTCTACTC	
miEBLN/X/P-1_F11	CAATGCACATACAGGAGTAAG	
miEBLN/X/P-1_F12	CTCTTCAGTGACTACTCCA	
miEBLN/X/P-2_F1	AGGTCATTTACTCCTCCATC	Sequencing of PCR-amplified miEBLN/X/P-2 locus region in M. fuliginosus
miEBLN/X/P-2_F2	GTCACTCTACAGTACCAACT	
miEBLN/X/P-2_F3	CAGGTTTCAAGGTTCCAGAT	
miEBLN/X/P-2_F4	GACCAGGAAATCGCATCTAT	
miEBLN/X/P-3_F0	CAGACCATCCTTTGTGGTTA	Sequencing of PCR-amplified miEBLN/X/P-3 locus region in M. fuliginosus
miEBLN/X/P-3_F1	GTGCGTGCTAGTGTTAGCT	
miEBLN/X/P-3_F2	GACGGTCAGGACATGGAA	
miEBLN/X/P-3_F3	CTCTAAGCCACCCTGGAT	
miEBLN/X/P-3_F4	GAGAGTCAAGGGGAGGTA	
miEBLN/X/P-3_F5	CTGTAGCTGCATCTTCATAC	
miEBLN/X/P-3_F6	CTGAGCTGGCAATCTAATC	
miEBLN/X/P-3_F7	CTTGTTGTGACGCCACTCA	
miEBLN/X/P-3_F8	CAATAAGTAGGCTTGCCCA	
miEBLN/X/P-3_F9	GTGTCAGCAACAATTGTGGA	
miEBLN/X/P-3_F10	GCACCAGATATTATGCTGAG	
miEBLN/X/P-3_R2	AACGGAGAGAAGATTCTCAC	
miEBLN/X/P-3_R6	TGTTCAACCGCGTCTTGAC	
miEBLX/P-4_F0	GTTAGGATGGAGTGGCAG	Sequencing of PCR-amplified miEBLX/P-4 locus region in *M. fuliginosus*
miEBLX/P-4_F1	AAGTTGCTTTAACTCTGGCTC	
miEBLX/P-4_F2	CTTGACTTGCTTGACAAAGTC	
miEBLX/P-4_R2	ATAGTACTTGTCCCAACCTG	
miEBLX/P-4_F3	GAGGAACTAAGCTCAAAGGA	
miEBLX/P-1_F4	CAGCATTATCTCTATACCCAG	
miEBLX/P-4_F5	GAAGAATTCTAGCAGGTGGTA	
miEBLX/P-4_F6	AACCGTAAAGTTTGTGGGAG	
miEBLX/P-4_R6	TATGGCAAGCTTTCAGTAGAC	
miEBLX/P-4_F7	GAAAGCTTGCCATATTGCTTTC	
pcDNA3-C-FLAG	TAACGGCCGCCAGTGTGCTGGAATTCGACGACTACAAGGACGACGA TGACAAGTGACTCGAGCATGCATCTAGAGGGCCCTA	Insertion of C-terminal FLAG tag sequence in pCDNA3
pcDNA3-C-MYC	TAACGGCCGCCAGTGTGCTGGAATTCGACGAACAAAAACTCATCTCA GAAGAGGATCTGTGACTCGAGCATGCATCTAGAGGGCCCTA	Insertion of C-terminal MYC tag sequence in pCDNA3
MFX-MYC_F	ATAGGGAGACCCAAGCTTGGTACCATGTCATCTGAAGAGAACAA	PCR amplification of MYC-tagged miEBLX-4 from *M. fuliginosus*
MFX-MYC_R	TGAGATGAGTTTTTGTTCGTCAGCCTGGTTTCCACCTCTG	
MFP-FLAG_F	ATAGGGAGACCCAAGCTTGGTACCATGTTGGGTCTCGAGGAA	PCR amplification of FLAG-tagged miEBLP-4 from *M. fuliginosus*
MFP-FLAG_R	TCATCGTCGTCCTTGTAGTCGTCATCAAGTACTAAATCACCTACAG	

Table S8. Accession numbers and regions used to identify orthologous EBLs and corresponding empty loci.

EBL	Species	Accession	Locus used for alignment		EBL region	
			Start	End	Start	End
miEBLN/X/P-1	*Miniopterus fuliginosus*	LC708266.1	1	7000	3841	5014
	Miniopterus natalensis	LDJU01000053.1	447562	440559	443717	441818
	Miniopterus schreibersii	PVJG01001939.1	118381	111364	114533	112625
	Phyllostomus hastatus	JAHKBD010000431.1	19156793	19162071	-	-
	Artibeus jamaicensis	VSFN01038119.1	1817044	1811801	-	-
	Eidolon helvum	AWHC01217387.1	86	5326	-	-
	Rhinolophus ferrumequinum	RXPC01000057.1	21846685	21851970	-	-
	Tadarida brasiliensis	PVIG010001649.1	78585	74262	-	-
	Myotis lucifugus	AAPE02008950.1	68899	67531	-	-
	Eptesicus fuscus	ALEH01008478.1	11058	12589	-	-
	Lasiurus borealis	PVJN01010870.1	39097	40660	-	-
miEBLN/X/P-2	*Miniopterus fuliginosus*	LC708265.1	-	-	1	1952
	Miniopterus natalensis	LDJU01000641.1	-	-	199193	197255
	Miniopterus schreibersii	PVJG01000137.1	-	-	102880	104818
miEBLN/X/P-3	*Miniopterus fuliginosus*	LC708264.1	1	4358	777	2671
	Miniopterus natalensis	LDJU01000514.1	755824	761474	756599	758498
	Miniopterus schreibersii	PVJG01014896.1	30962	25318	30182	28293
	Myotis lucifugus	AAPE02020528.1	59349	63680	-	-
	Murina aurata	PVJC01020493.1	23338	27450	-	-
	Hipposideros turpis	JAHQIX010000004.1	1773895	1781831	-	-
	Nycticeius humeralis	VMDQ010008967.1	24649	17787	-	-
	Rhinolophus ferrumequinum	VMDN01008025.1	43577	35740	-	-
	Molossus molossus	JACASF010000017.1	58511808	58506690	-	-
miEBLX/P-4	*Miniopterus fuliginosus*	LC708263.1	1	3256	1392	2216
	Miniopterus natalensis	LDJU01000157.1	191495	194755	192892	193714
	Miniopterus schreibersii	PVJG01006939.1	46782	43522	45386	44562
	Pteropus giganteus	CACVBW010014110.1	28243464	28241025	-	-
	Rousettus aegyptiacus	BNJL01000102.1	75462725	75460270	-	-
	Tadarida brasiliensis	PVIG010005733.1	56729	59446	-	-
	Myotis lucifugus	AAPE02022042.1	34740	37356	-	-
	Pipistrellus kuhlii	JACAGB010000005.1	62995611	62992837	-	-
	Antrozous pallidus	VMDP01000463.1	54501	50831	-	-
	Phyllostomus hastatus	JAHKBD010000164.1	18943190	18945427	-	-
	Artibeus jamaicensis	VSFN01055018.1	2001167	2003528	-	-
mpEBLX/P	*Phyllostomus hastatus*	JAHKBD010000169.1	433470	437868	435164	435957
	Phyllostomus discolor	RXPB02010240.1	189957	194322	191633	192427
	Macrotus californicus	VMDR010008610.1	15713	11334	14035	13238
	Carollia perspicillata	PVKM010001206.1	22167	26583	23834	24646
	Desmodus rotundus	PEHR01051769.1	1737	5855	3178	3937
	Artibeus jamaicensis	PVKR01001943.1	35051	39411	36721	37538
	Micronycteris hirsuta	PVJI01003473.1	93967	98090	95418	96182
	Tonatia saurophila	PVIA01006371.1	94105	98321	95574	96365
	Anoura caudifer	PVKU01001816.1	130645	135015	132365	133164
	Sturnina hondurensis	VSFL01024317.1	281229	285166	282727	283477
	Pteronotus parnellii	AWGZ01068455.1	2684	6602	4132	4933
	Mormoops blainvillei	PVJD01000578.1	284124	280109	282643	281883
	Miniopterus natalensis	LDJU01000038.1	3474535	3477727	-	-
	Noctilio leporinus	PVIW01002060.1	79119	75674	-	-
	Tadarida brasiliensis	PVIG010004157.1	41121	44632	-	-
	Eidolon helvum	AWHC01203757.1	4332	1599	-	-
	Rhinolophus ferrumequinum	RXPC01000007.1	73068418	73072351	-	-
ctEBLX/P	*Craseonycteris thonglongyai*	PVKE010012405.1	1507	9684	3230	7142
	Miniopterus natalensis	LDJU01000019.1	870930	874292	-	-
	Megaderma lyra	PVJL010002460.1	177034	180262	-	-
	Pteropus giganteus	CACVBW010009929.1	25041708	25044962	-	-
	Rousettus aegyptiacus	JACASE010000002.1	111694139	111690883	-	-
	Myotis lucifugus	AAPE02006437.1	165488	169250	-	-
	Artibeus jamaicensis	VSFN01032908.1	852299	848076	-	-
	Phyllostomus hastatus	JAHKBD010000216.1	330081	336301	-	-
ocEBLX/P	*Octodon degus*	AJSA01129841.1	5835	12024	9920	10638
	Octomys mimax	NDGM011201982.1	4578	1857	4035	3306
	Tympanoctomys barrerae	NDGN011162872.1	1	3398	2295	3015
	Myocastor coypus	PVJA010015660.1	18690	24538	22551	23274
	Ctenomys sociabilis	PVKA01016282.1	19345	25783	23185	24430
	Capromys pilorides	PVKN010358143.1	1	1712	275	996
	Chinchilla lanigera	AGCD01044698.1	35788	30939	-	-

Erethizon dorsatum	SWEC01019031.1	181322	176127	-	-
Dinomys branickii	PVLD010018757.1	16974	21844	-	-
Heterocephalus glaber	RPGA01000030.1	8476243	8470892	-	-
Hystrix cristata	PVJO010015485.1	10119	5355	-	-
Hydrochoerus hydrochaeris	JADBBS010000005.1	58570690	58566082	-	-

Table S9. Accession numbers of orthobornaviruses used in this study.

Virus	Abbreviation	Accession
Borna disease virus 1	BoDV-1	AJ311522
Borna disease virus 2	BoDV-2	AJ311524
Variegated squirrel bornavirus 1	VSBV-1	LN713680
Aquatic bird bornavirus 1	ABBV-1	KF578398
Canary bornavirus 1	CnBV-1	KC464471
Parrot bornavirus 2	PaBV-2	EU781967
Loveridge’s garter snake virus 1	LGSV-1	KM114265
Caribbean watersnake bornavirus	CWBV	BK014571
