# Supplementary material for: The hidden diversity of ancient bornaviral sequences from X and P genes in vertebrate genomes
Source: Virus Evol. 2023 Jun 3;9(1):vead038. doi: 10.1093/ve/vead038 (PMC10288550; doi:10.1093/ve/vead038)
Supplement: vead038_Supp [file vead038_supp.zip › Figs_supp.pdf]

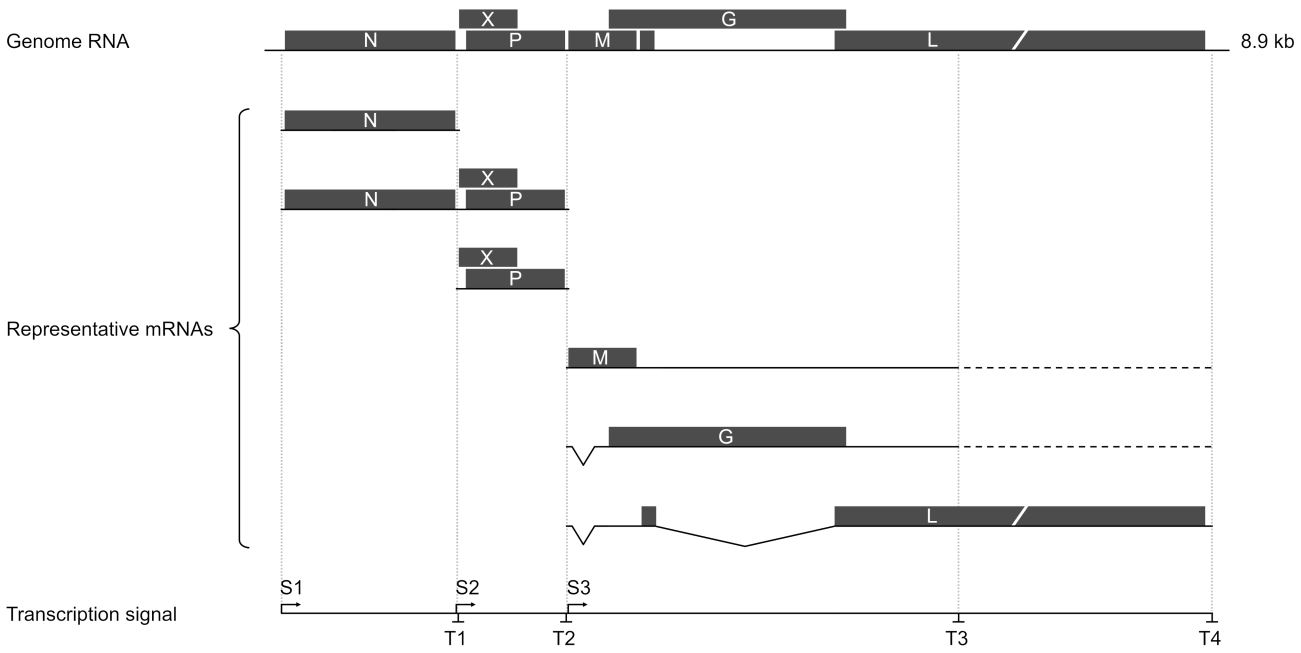

Supplementary Figure 1. Genome organization and transcripts of orthobornaviruses.
The viral genome RNA encodes six genes: N, X, P, M, G, and L. Viral mRNAs are transcribed by the L protein, according to transcription start (S1/S2/S3) and termination (T1/T2/T3/T4) signals within the viral genome RNA. Notably, the N/X/P mRNA can be produced as byproduct of read-through transcription at T1 and subsequent termination at T2.

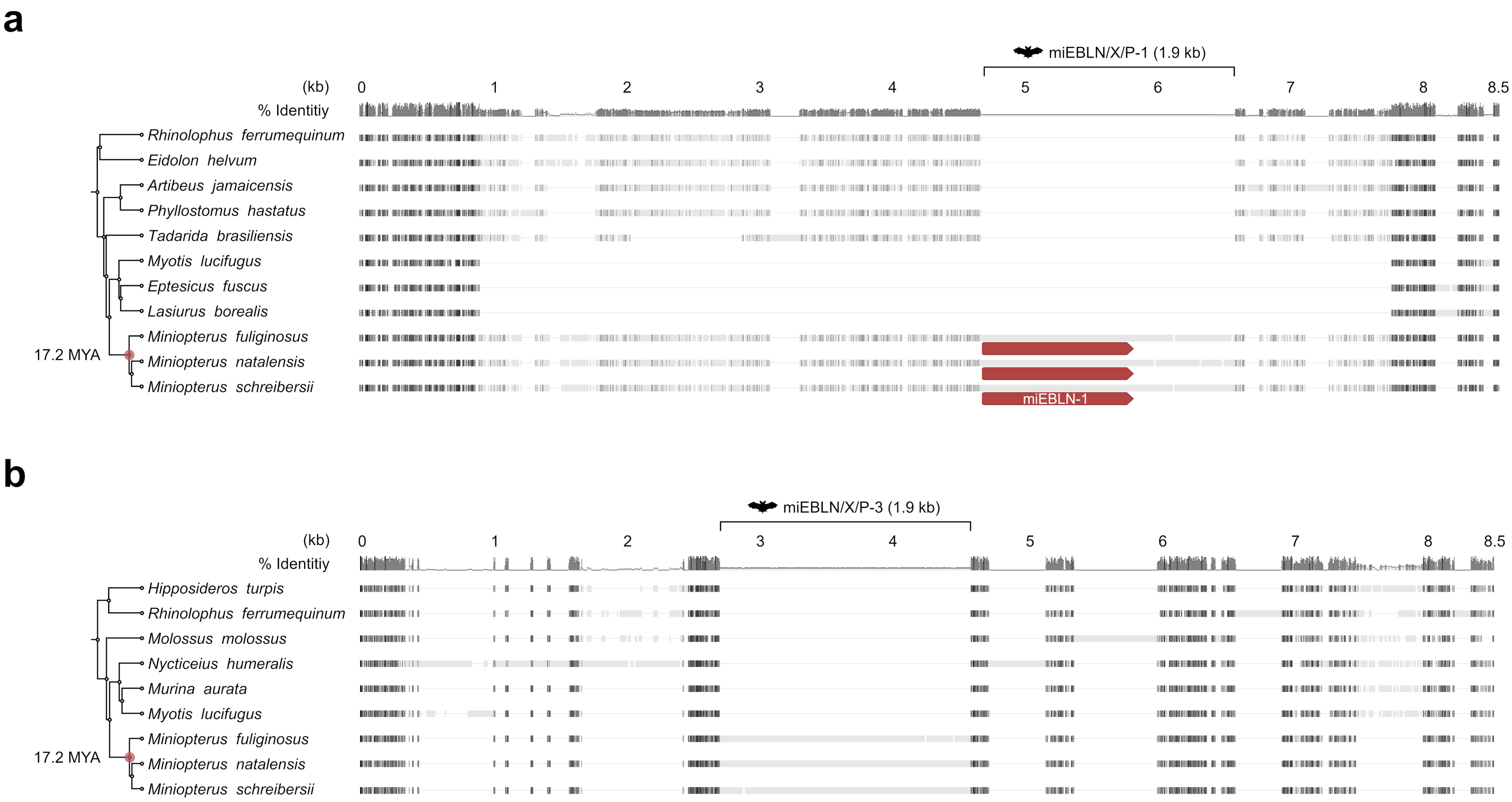

Supplementary Figure 2. Gene orthology analysis of EBLN/X/P-1 and EBLN/X/P-3.
Nucleotide sequence alignment of **(a)** miEBLN/X/P-1 and **(b)** miEBLN/X/P-3, and corresponding flanking loci are shown. Black boxes indicate conserved residues. Horizontal gray lines show alignment gaps. Species phylogeny and deduced minimum integration age of each EBLN/X/P are indicated on the left based on TimeTree (1). Red arrow boxes indicate miEBLN-1 ORF.

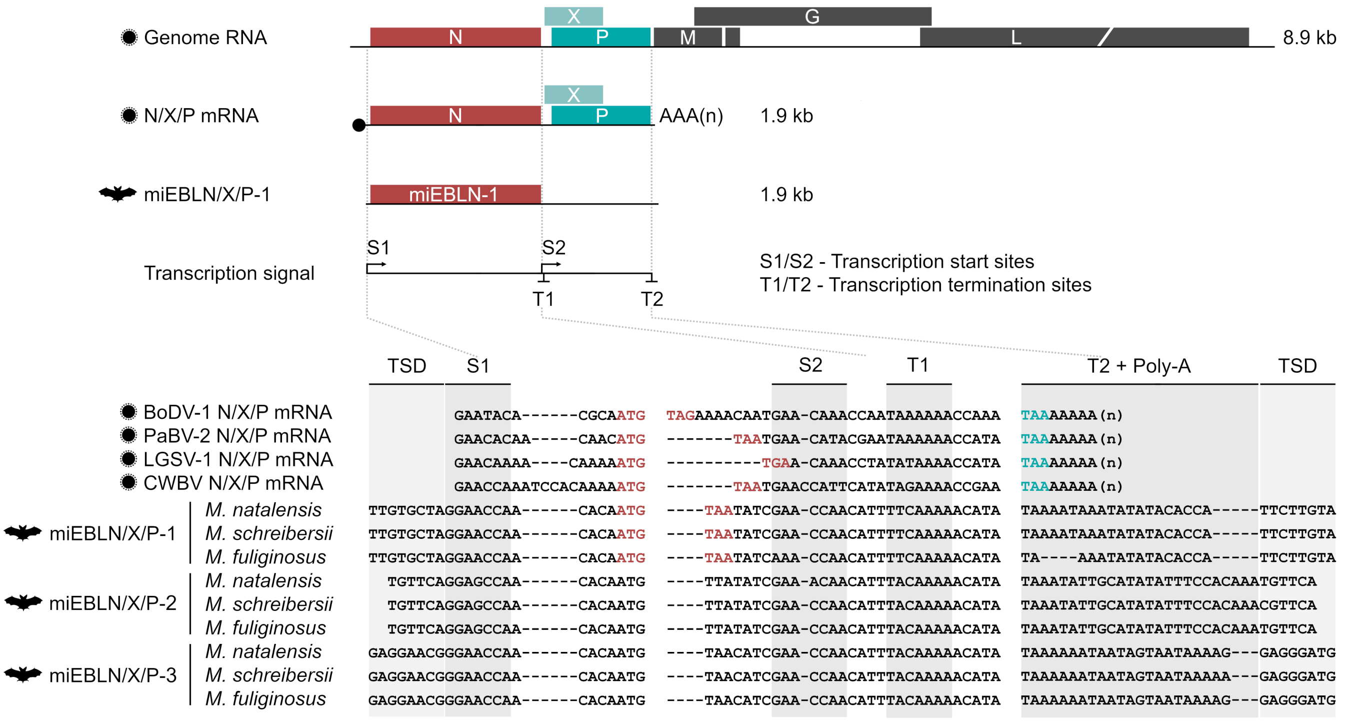

Supplementary Figure 3. Nucleotide sequence alignment of extant orthobornaviral N/X/P transcripts and EBLN/X/Ps.

Upper panel shows the schematic diagram of orthobornaviral genomic RNA, 1.9-kb N/X/P mRNA, and miEBLN/X/P-1, and location of transcription signals (S1, S2, T1, and T2). Lower panel shows the nucleotide sequence alignment of orthobornaviral 1.9-kb N/X/P mRNA and miEBLN/X/P-1, -2, and -3. Red letters indicate the start and stop codons of the N gene. Teal blue letters indicate the the stop codon of P gene. TSD, target site duplication.

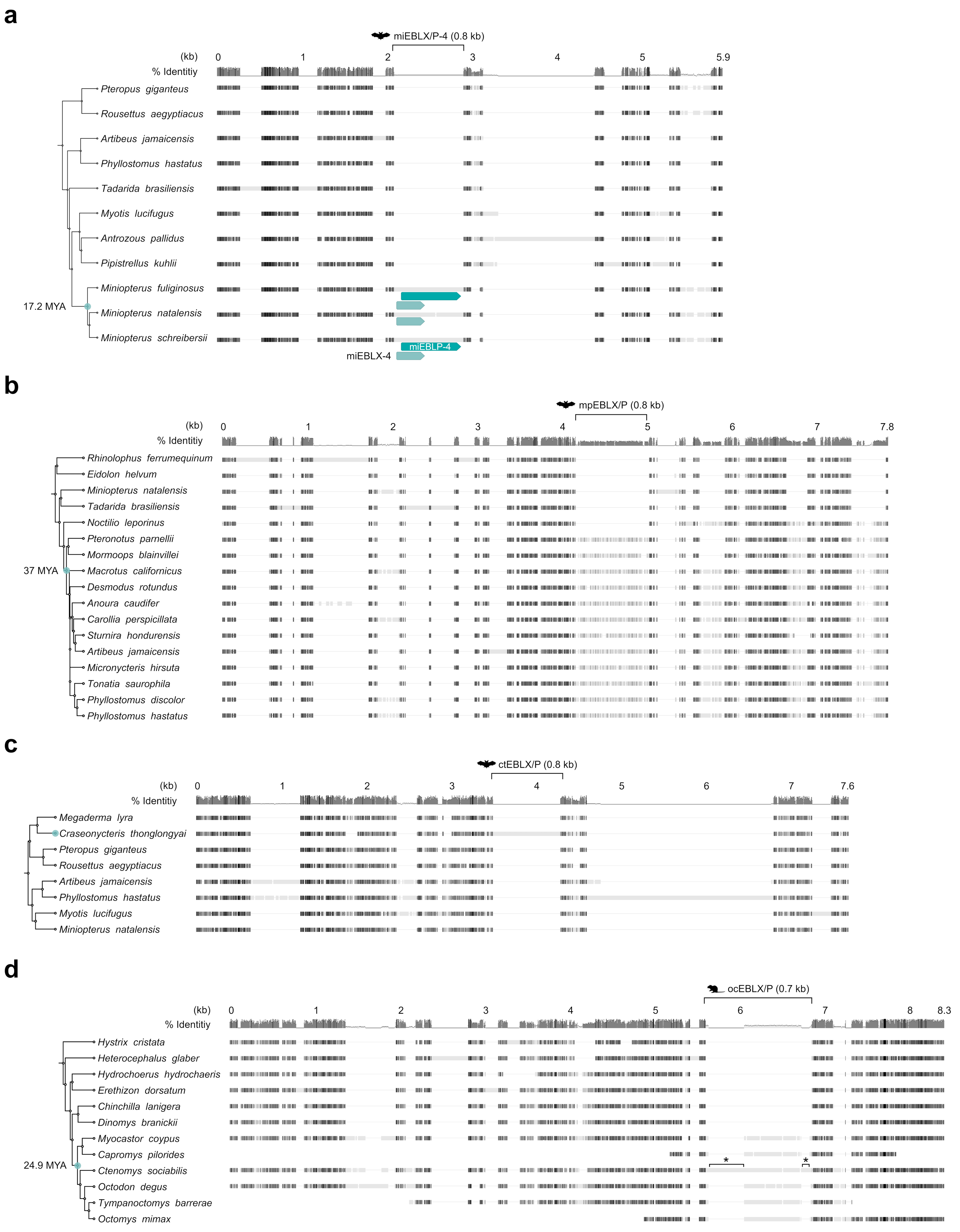

Supplementary Figure 4. Gene orthology analyses of EBLX/Ps.
Nucleotide sequence alignment of **(a)** miEBLX/P-4, **(b)** mpEBLX/P, **(c)** ctEBLX/P, and **(d)** ocEBLX/P, and corresponding flanking loci are shown. Black boxes indicate conserved residues. Horizontal gray lines show alignment gaps. Species phylogeny and deduced minimum integration age of each EBLX/P are indicated on the left based on TimeTree (1). Light and dark teal blue arrow boxes indicate miEBLX-4 and miEBLP-4 ORFs, respectively. *ocEBLX/P in *C. sociabilis* contains insertions.

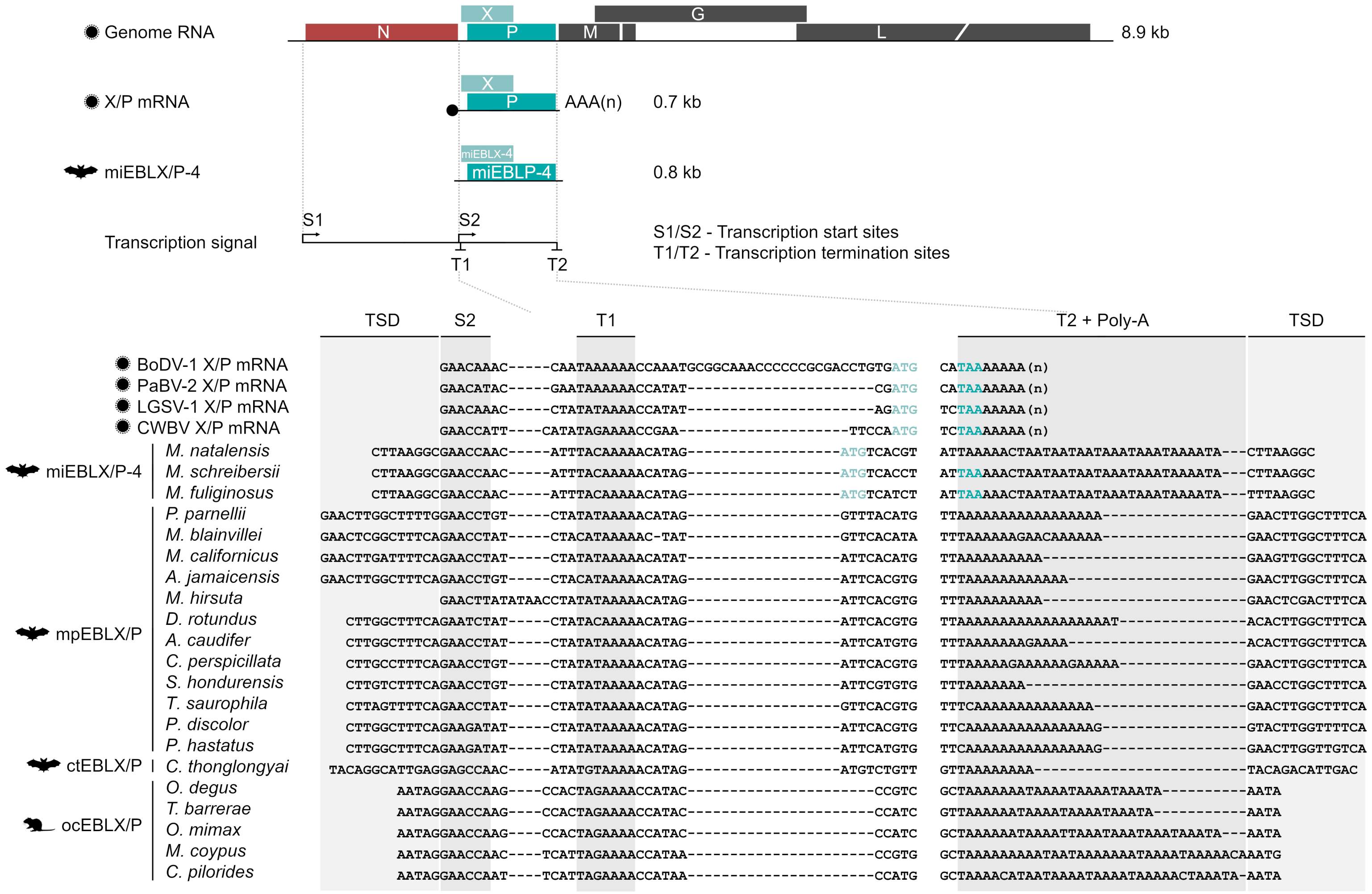

Supplementary Figure 5. Nucleotide sequence alignment of extant orthobornaviral X/P transcripts and EBLX/Ps.

Upper panel shows the schematic diagram of orthobornaviral genomic RNA, 0.7-kb X/P mRNA, and miEBLX/P-4, and location of transcription signals (S1, S2, T1, and T2). Lower panel shows the nucleotide sequence alignment of orthobornaviral 0.7-kb X/P mRNA, and miEBLX/P-4, mpEBLX/P, ctEBLX/P, and ocEBLX/P. Light and dark teal blue letters indicate the start codon of X gene and the stop codon of P gene, respectively. TSD, target site duplication.

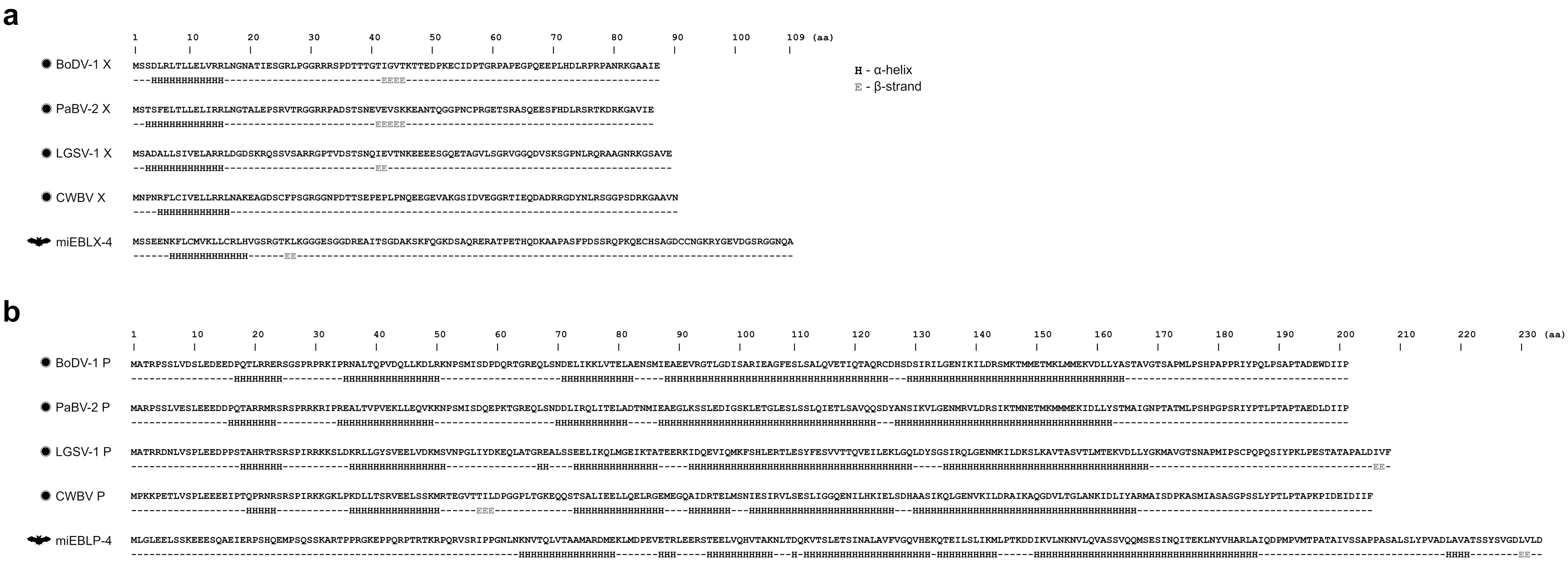

Supplementary Figure 6. Secondary structure prediction of putative EBLX and EBLP proteins.
Predicted secondary structure of **(a)** extant orthobornaviral X and miEBLX-4 proteins, and **(b)** extant orthobornaviral P and miEBLP-4 proteins. The analyzed viruses were selected as representative from each clade of the orthobornavirus phylogenetic tree (2). Black H: α-helix. Gray E: β-strand.

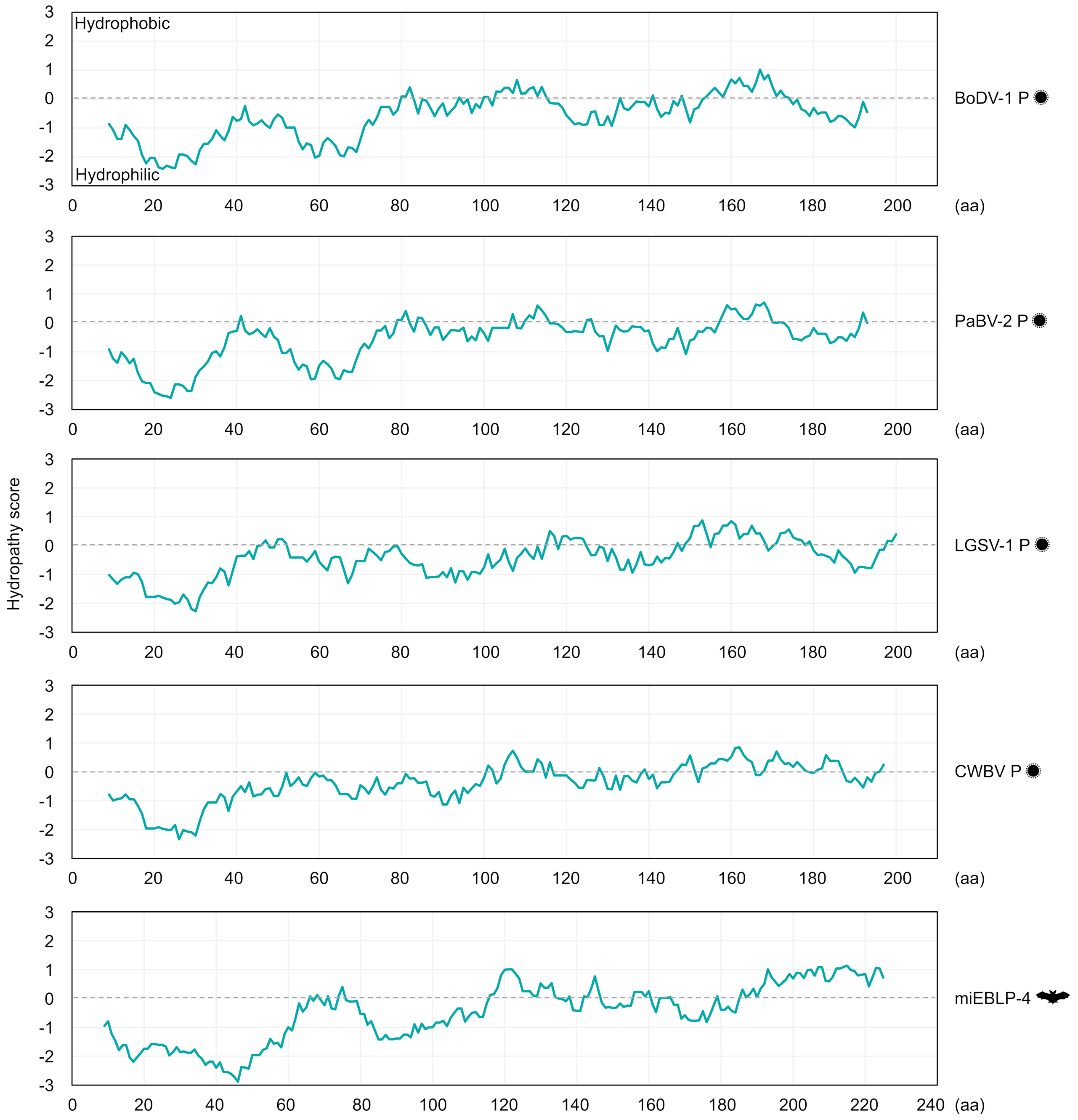

Figure S7. Hydrophobicity of putative EBLP protein.
Hydropathy scores were plotted along the amino acid sequences of extant orthobornaviral P and miEBLP-4 proteins. Positive scores indicate hydrophobic residues while negative scores indicate hydrophilic residues.

References

1. Kumar S, Stecher G, Suleski M, Hedges SB. TimeTree: A Resource for Timelines, Timetrees, and Divergence Times. Mol Biol Evol. 2017;34(7):1812-9.

2. Rubbenstroth D, Briese T, Durrwald R, Horie M, Hyndman TH, Kuhn JH, et al. ICTV Virus Taxonomy Profile: Bornaviridae. J Gen Virol. 2021;102(7).
